# Plasmon resonance of gold and silver nanoparticle arrays in the Kretschmann (attenuated total reflectance) vs. direct incidence configuration

**DOI:** 10.1038/s41598-022-20117-7

**Published:** 2022-09-21

**Authors:** Rituraj Borah, Rajeshreddy Ninakanti, Sara Bals, Sammy W. Verbruggen

**Affiliations:** 1 Sustainable Energy, Air and Water Technology (DuEL)Department of Bioscience Engineering, University of Antwerp, Groenenborgerlaan 171, 2020 Antwerp, Belgium; 2 Electron Microscopy for Material Science (EMAT), Department of Physics, University of Antwerp, Groenenborgerlaan 171, 2020 Antwerp, Belgium; 3 NANOlab Center of Excellence, University of Antwerp, Groenenborgerlaan 171, 2020 Antwerp, Belgium

**Keywords:** Nanoparticles, Two-dimensional materials, Nanophotonics and plasmonics, Nanophotonics and plasmonics

## Abstract

While the behaviour of plasmonic solid thin films in the Kretschmann (also known as Attenuated Total Reflection, ATR) configuration is well-understood, the use of discrete nanoparticle arrays in this optical configuration is not thoroughly explored. It is important to do so, since close packed plasmonic nanoparticle arrays exhibit exceptionally strong light-matter interactions by plasmonic coupling. The present work elucidates the optical properties of plasmonic Au and Ag nanoparticle arrays in both the direct normal incidence and Kretschmann configuration by numerical models, that are validated experimentally. First, hexagonal close packed Au and Ag nanoparticle films/arrays are obtained by air–liquid interfacial assembly. The numerical models for the rigorous solution of the Maxwell’s equations are validated using experimental optical spectra of these films before systematically investigating various parameters. The individual far-field/near-field optical properties, as well as the plasmon relaxation mechanism of the nanoparticles, vary strongly as the packing density of the array increases. In the Kretschmann configuration, the evanescent fields arising from *p*- and *s*-polarized (or TM and TE polarized) incidence have different directional components. The local evanescent field intensity and direction depends on the polarization, angle of incidence and the wavelength of incidence. These factors in the Kretschmann configuration give rise to interesting far-field as well as near-field optical properties. Overall, it is shown that plasmonic nanoparticle arrays in the Kretschmann configuration facilitate strong broadband absorptance without transmission losses, and strong near-field enhancement. The results reported herein elucidate the optical properties of self-assembled nanoparticle films, pinpointing the ideal conditions under which the normal and the Kretschmann configuration can be exploited in multiple light-driven applications.

## Introduction

Ordered arrangement of nanoparticles into periodic arrays over large areas is an important requirement to obtain functional interfaces for real-life applications. When plasmonic nanostructures are arranged periodically, electrodynamic coupling of the nanostructures enhances the light-matter interaction as compared to isolated nanostructures^1,2^. Thus, periodic arrays of nanostructures have gained increasing attention in recent years for the application in light driven processes such as solar energy harvesting^3^, photovoltaics^4^, photocatalysis^5,6^, SERS (surface-enhanced Raman spectroscopy)^7^, fluorescence^8^, SEIRA (surface-enhanced infrared absorption spectroscopy)^9^, sensing^10^, photoemission and photodetection^11^, etc. The plasmonic enhancement of these various processes can occur through different mechanisms. For instance, for energy harvesting applications, the absorption intensity of individual nanostructures, i.e. the far-field optical properties, are more important than the near-field enhancement. In contrast, surface enhanced processes such as SERS, SEIRA, surface enhanced fluorescence^12,13^, etc., strongly rely on the near-field enhancement effect. Refractive index-based plasmonic sensing is based on the changes in the far-field characteristics due to changes in the dielectric properties in the near-field region^14^. In plasmon-enhanced photocatalysis, a dual effect of both plasmonic hot electron injection and near-field enhancement is believed to be driving the enhancement mechanism^15^. The plasmonic coupling in periodic arrays of nanoparticles has important implications in all of the above-mentioned applications, as the individual optical properties of nanoparticles in an array can be dramatically different from those of isolated nanoparticles.

Among different ways to fabricate periodic arrays of nanostructures, self-assembly is a facile and cost-efficient emerging technique that has been gaining increasing attention^16^. Self-assembly is a bottom up approach where the nanoparticles are the building blocks forming the 1D, 2D or 3D arrays. The air–liquid interfacial assembly is a common self-assembly technique that can be utilized to obtain nanoparticle arrays^17^. The interparticle gap in air–liquid interfacial assembly can be controlled by strategies such as changing the ligand chain length^18,19^, or surface pressure regulation^20^. Other common self-assembly techniques such as evaporative self-assembly or directed self-assembly on functionalized substrates are also effective strategies^21^. Alternatively, Bansmann et al. demonstrated an in-situ nanoparticle synthesis technique by the reduction of Au precursor contained in periodically arranged reverse micelles for excellent control over the interparticle gap^22^.

In view of the growing importance of self-assembled plasmonic nanoparticle arrays and their potential applications, a thorough understanding of their optical properties is key^1^. Apart from the material properties of the array, also the irradiance mode plays a pivotal, yet less well understood role in driving the optical properties of such films. Next to the traditional direct normal incidence of light on the nanoparticle arrays, the ATR (attenuated total reflection) also known as the Kretschmann configuration is an interesting optical configuration that is already quite common in sensing applications^23^. While this Kretschmann configuration is commonly applied in combination with thin planar films, the use of discrete plasmonic nanoparticles and nanoparticle arrays in this configuration has hardly been explored. Therefore, in this work, the optical properties of self-assembled arrays of Au and Ag nanoparticles in direct normal incidence and Kretschmann (i.e*.* ATR) configuration are investigated by numerical models. First, close packed colloidal Au and Ag nanoparticle films were obtained by air–ethylene glycol interfacial assembly for the validation of the models by the comparison of experimental and computed spectra. The optical properties of the Au and Ag nanoparticle arrays are then studied in terms of their far-field and near-field optical properties, when varying the interparticle gap, type of substrate, optical configuration, and polarization direction of the incoming electromagnetic wave. The individual optical properties of nanoparticles in close packed arrays and when they are isolated are compared as well.

## Materials and methods

### Synthesis of Au nanoparticles and phase transfer

Au nanoparticles were synthesized by the well-known citrate reduction method. A 100 mL 5 mM tri-sodium citrate solution in water was brought to boil before the addition of 1 mL of 25 mM HAuCl_4_ solution. Following the precursor addition, the solution was kept boiling for 20 more minutes for complete reduction. The as-synthesized nanoparticles were centrifuged at 11,529*g* for 30 min and redispersed in 10 mL Milli-Q water to obtain a 10 times more concentrated colloidal solution. The concentrated Au nanoparticles in the aqueous phase were transferred to a toluene phase by using oleylamine as a hydrophobic ligand. 3 mL of toluene were gently added onto the surface of 1 mL of aqueous Au nanoparticle colloid so that the toluene floats over the water as a separate phase. To this bi-phasic liquid, 2 mL of oleylamine in ethanol solution (200 mg in 20 mL ethanol) were added followed by gentle shaking for mixing and then resting for separation of the toluene and ethanol–water phases. After resting the mixture for 24 h, the nanoparticles appear in the toluene phase above (indicated by a distinct red color) and the ethanol–water phase below becomes transparent. The nanoparticles in the toluene phase were then carefully pipetted out for further processing.

### Synthesis of Ag nanoparticles and phase transfer

Au nanoparticles were synthesized by the well-known citrate reduction method in presence of tannic acid as reducing agent, as reported by Bastus et al.^24^. A 100 mL 5 mM tri-sodium citrate and 0.1 mM tannic acid solution in water was brought to a boil before the addition of 1 mL of 25 mM AgNO_3_ solution. Following the precursor addition, the boiling of the solution was continued for 20 more minutes for complete reduction of the Ag ions. The as-synthesized nanoparticles were concentrated 10 times by centrifuging at 11,529*g* for 20 min and re-dispersing in milli-Q water. The concentrated Ag nanoparticles in the aqueous phase were transferred to the toluene phase by a procedure similar to Au nanoparticles as described above. A higher concentration of oleylamine in ethanol (100 mg in 20 mL ethanol) was used in this case. Also, the oleylamine solution was added first to the nanoparticle colloid before the addition of the toluene phase and mixing.

### Self-assembly

**Figure 1:**
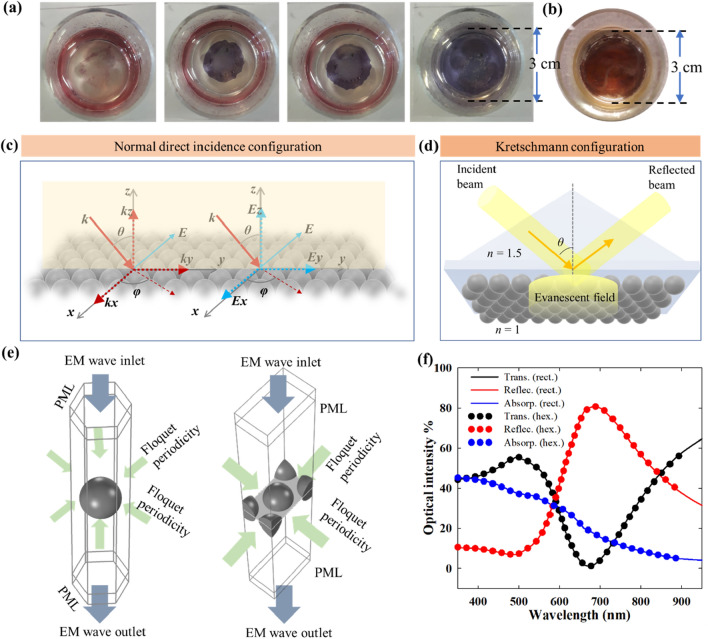
(**a**) Development of the nanoparticle film during the self-assembly of Au nanoparticles over ethylene glycol surface for 8 h. (**b**) Self-assembled Ag nanoparticle film after solvent evaporation for 8 h. (**c**) Schematic representation of a self-assembled nanoparticle monolayer with incident and electric field wave vectors w.r.t. the 3-D cartesian axes. The incident angle *θ* is varied only for the Krestchmann i.e. ATR configuration as discussed later. (**d**) Schematic representation of plasmonic nanoparticle film in the Kretschmann configuration. (**e**) Hexagonal and rectangular unit cells with periodic boundary conditions implemented as the computational domains in the electromagnetic modeling. (**f**) Comparison of the results obtained from rectangular and hexagonal computational domains.

The same self-assembly procedure was adopted for both Au and Ag nanoparticles. The nanoparticles in toluene after phase transfer (2 mL) were concentrated by centrifugation at 10,625*g* for 20 min to 200 µL and then introduced onto the surface of ethylene glycol contained in a glass container of 30 mm in diameter. The beaker was then immediately covered by a glass plane, allowing toluene to evaporate slowly (Fig. 1a,b). After the complete evaporation of toluene (in 8 h), the nanoparticle films were transferred to the substrates for further characterization and application. The nanoparticle films were transferred to a glass substrate by careful vertical dip coating similar to the Langmuir–Blodgett technique. The films were dried under vacuum at 40 °C for 48 h. For bilayer films, two films were deposited sequentially one on the other.

### Characterization

The as-obtained films on glass were characterized by UV–Vis spectroscopy in transmission mode, using a Shimadzu UV2600 apparatus. For the bright field transmission electron microscopy (TEM) of the self-assembled nanoparticles, a FEI Tecnai G2 operated at an accelerating voltage of 200 kV was used. To prepare the TEM samples, the nanoparticle films were immobilized on a hydrophobic copper grid by dip coating before vacuum drying at 100 °C for 12 h.

### Electromagnetic modelling

The computational electromagnetic models for the rigorous solution of the Maxwell’s equations are built on a FEM solver in COMSOL Multiphysics (see supporting information). As shown in Fig. 1c, an infinite film of close packed nanoparticles is incident on by an incoming wave in the normal direct incidence configuration. The polarization of the incident wave is determined by the angle of incidence as well as the direction of the electric field. For a fixed *θ*, *φ* = 0° forms the *s*-polarization and *φ* = 90° forms the *p*-polarization. For the cases with the direct incidence configuration, the incidence is normal i.e. *θ* = 0°. The polarizations at normal incidence is then defined by *φ* as shown in Figs. 7, 8 and 9. In Fig. 1d, the Kretschmann (ATR) configuration is shown where the incident beam comes from a high refractive index medium, i.e. the prism, and the nanoparticle film (or any substance of interest being probed) is on the other side where the medium has a lower refractive index. The incoming beam is incident at an angle higher than the critical angle for the given set of refractive indexes, so that it is reflected by total internal reflection at the interface. Thus, solely the resulting standing evanescent wave on the lighter medium interacts with the object of interest. Properties of the Kretschmann configuration are discussed further in later sections.

For the electromagnetic modeling, the infinite 2D arrays were approximated by a unit cell with periodic boundary conditions on the side walls as shown in Fig. 1e. The incident electromagnetic field was introduced from the top with the wave outlet at the bottom for the full-field solution. A perfectly matched layer (PML) was implemented on the top and the bottom boundaries to ensure total absorption of the propagating wave. To ensure the accuracy of the periodic boundary conditions, both rectangular and hexagonal unit cell models were constructed for comparison (Fig. 1f). To ensure the mesh and the domain independence of the numerical results, mesh and domain independence tests were performed with varying mesh elements and domain sizes. The computational domains and the meshing scheme in the rectangular and hexagonal unit cell are shown in Figs. S1 and S2.

## Results and discussions

### Characterization of the films and computational validation

**Figure 2:**
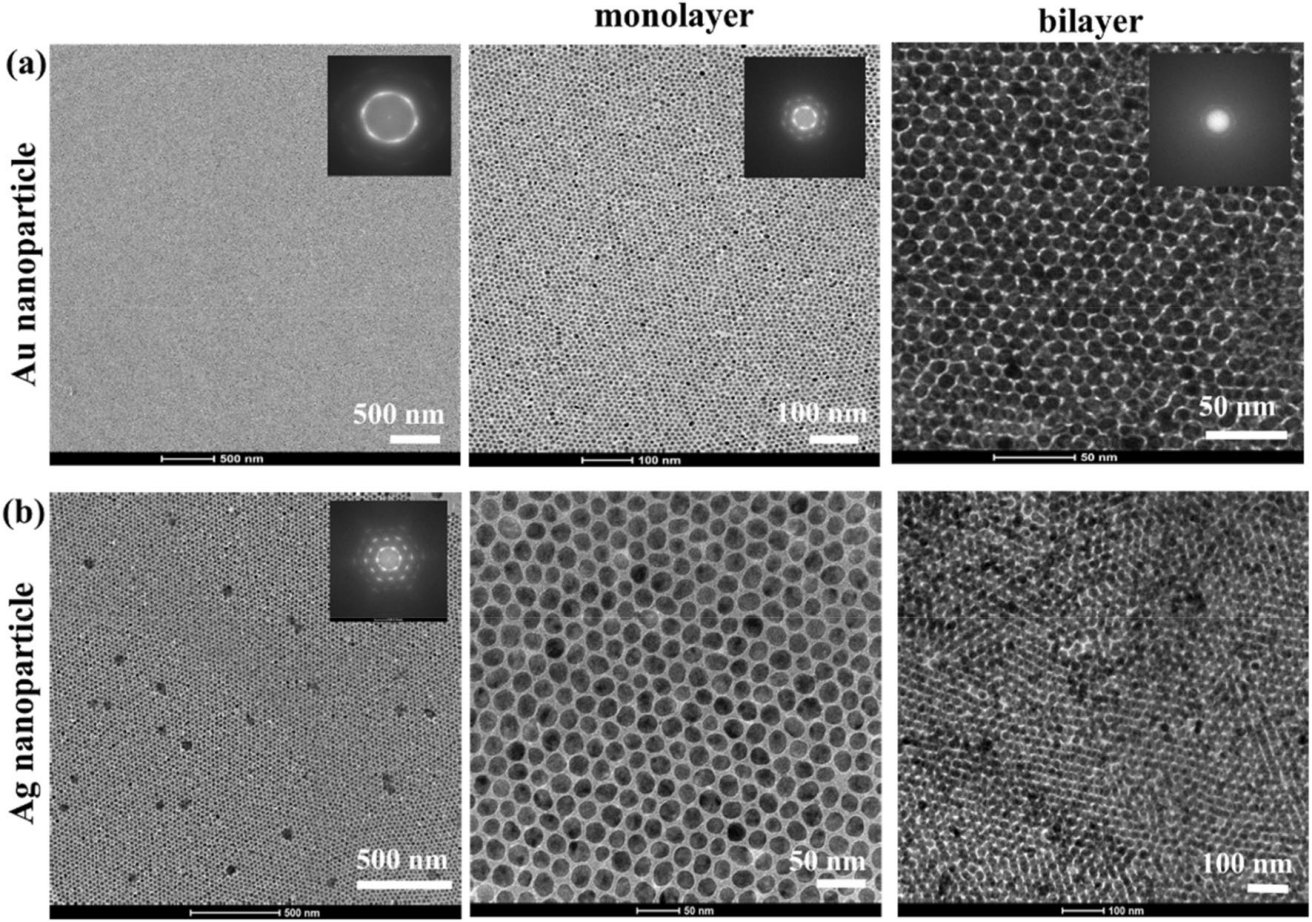
(**a**) TEM images of Au (**a**) and Ag (**b**) nanoparticle self-assemblies at different length scales.

Figure 2a,b show the self-assembled monolayer films of Au and Ag nanoparticles, respectively. The overall packing order of the films is clear from the fast Fourier transformations (FFT) of the images. The average sizes of the nanoparticles were estimated to be 10 ± 2 nm with an interparticle gap of ~ 2.1 nm for Au and 21 ± 3 nm with an interparticle gap of ~ 3 nm for Ag nanoparticles (Figs. S3 and S4). Overall, the particles are well-organized in an hexagonal close packed structure, while some polycrystalline domains with local defects and irregular particle morphology can be discerned. It has been shown that the presence of excess free ligands and excess nanoparticles (i.e. more nanoparticles than needed to cover the complete surface with a monolayer) enhance the degree of ordering during the self-assembly process^25,26^. Thus, after the water-to-toluene phase transfer, the nanoparticle colloids were concentrated to the desired concentration for self-assembly without further washing steps so that a certain amount of excess free ligands remained in the colloid to facilitate the ordering during self-assembly. As the number of Au nanoparticles (~ 2 × 10^13^) introduced over the ethylene glycol surface was much higher (> 4 times) than the number required (~ 5 × 10^12^) for covering the total surface area available, the excess nanoparticles exert pressure on the film for close packing. For this concentration of nanoparticles, the excess nanoparticles accumulate at the outer boundary of the film resulting in a predominantly monolayer film. A 3–4 times higher concentration on the other hand, leads to the formation of large bilayer areas (Fig. 2a,b). In order to ensure a uniform bilayer, deposition of one monolayer over another is a more effective strategy.

For the electromagnetic modeling of the self-assembled films, the average values of the nanoparticle diameter and interparticle distance in a unit cell with a hexagonal close packing were used. As shown by Schatz and co-worders, the irregularities in the 2D lattice such as polycrystallinity, small variations in the particle diameter and the interparticle gap do not lead to any dramatic changes in the optical properties^27^. Thus, average lattice parameters are sufficient to describe the optical properties of the films by electromagnetic models. The experimental transmittance spectra of the monolayer and bilayer nanoparticle films were measured after immobilization of the films on glass followed by vacuum drying at 40 °C for 12 h. Thus, the refractive index of the medium was assumed to attain a constant value of 1.4 to take into account the ethylene glycol still present in the film^28^. The optical constants for Au and Ag were taken from Johnson and Christy^29^, and Palik^30^ respectively. While optical constants of Au reported in various literature have a general agreement, for Ag, there exist some variations, as discussed in detail in earlier work. The suitability of the optical constants of Palik in predicting plasmonic response of colloidal nanoparticles has been established by taking into consideration all the influencing factors such as particle polydispersity, skewness of size distribution, morphological irregularity, and so on^31^. It is known that for particles of size 10 nm and larger, the quantum effects are not strong^32^. Barrow et al. showed that even for interparticle distances as small as 0.5 nm, there is still good agreement between classical electromagnetic calculations and experimental spectra as quantum tunnelling effects are not significant^33^. As the individual nanoparticles in our study are at least 10 nm or larger, and are separated by interparticle gaps of more than 1 nm, the classical computations can be relied upon.

**Figure 3:**
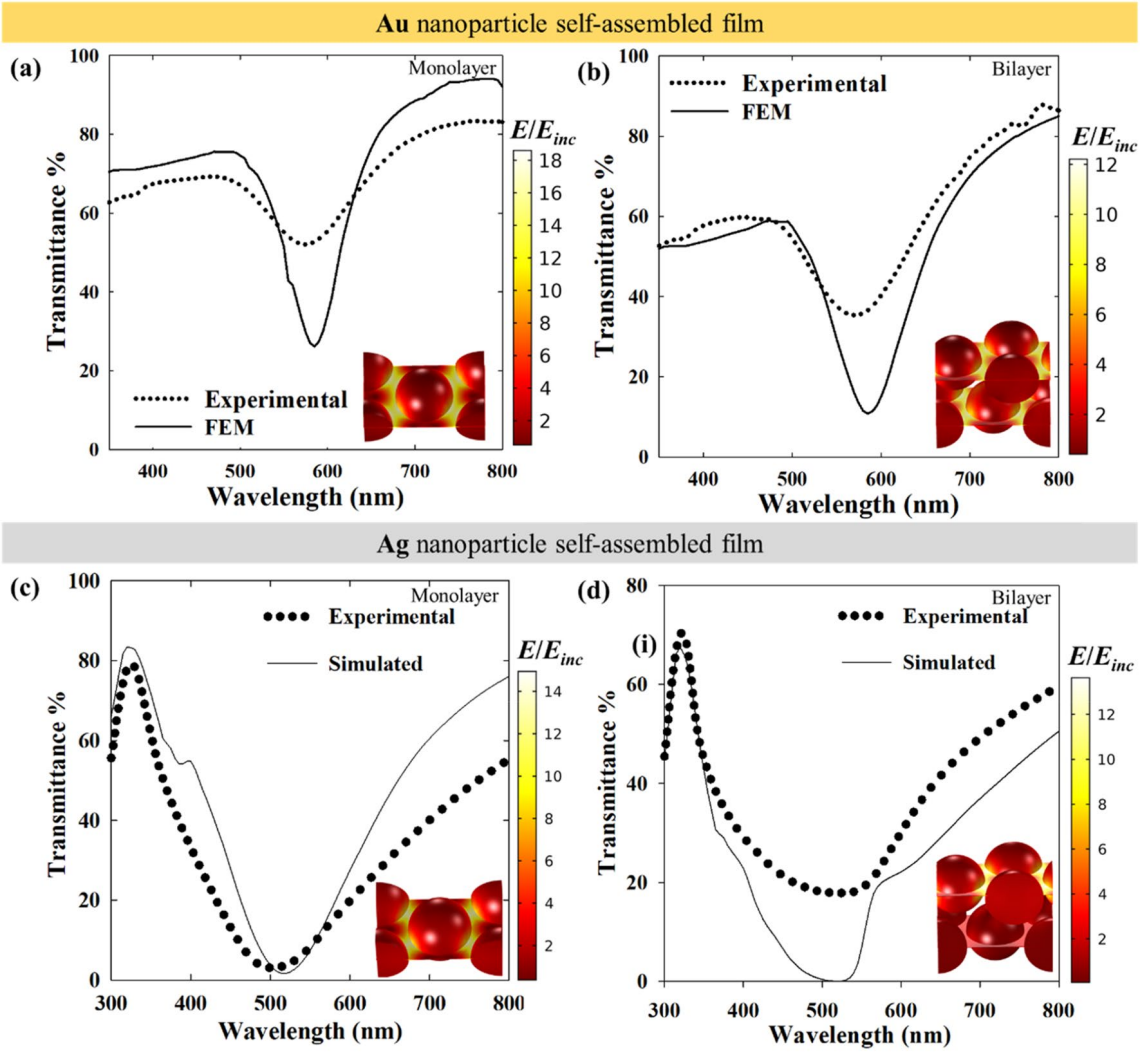
Experimental transmittance spectra compared with computed spectra for (**a**) Au nanoparticle monolayer, (**b**) Au nanoparticle bilayer, (**c**) Ag nanoparticle monolayer, (**d**) Ag nanoparticle bilayer. The near-field enhancement with respect to the incident field is shown in the inset of each plot. The dimensions for the models were taken from the TEM images (Fig. 2). Au nanoparticle diameter and interparticle gap: 10 nm and 2 nm, Ag nanoparticle diameter and interparticle gap: 20 and 2 nm. The direction of incidence of illumination is normal in all cases.

Figure 3 shows that the computed spectra largely match with the experimental spectra for all cases with respect to the lattice resonance positions and spectral features. The plasmon bands at ~ 600 and ~ 500 nm for Au and Ag nanoparticle films, respectively, are significantly red-shifted from their respective LSPR positions at ~ 532 and ~ 408 nm when isolated, confirming our findings for smaller clusters in a previous study^2^. Also, there is no significant difference in the lattice resonance position for monolayer and bilayer configurations. The near-field enhancement plots in the insets show that the films exhibit lattice resonance by strong near-field coupling. As small nanoparticles have low scattering intensity, far-field coupling is negligible or very low in these cases. For both Au and Ag, the near-field enhancement is stronger for monolayers as compared to bilayers. The suppressing effect of the lower layer of the bilayer stems from the reflection from the lower layer that interferes destructively with the field around the top layer^1^. Since the top layer of the Ag nanoparticle bilayer is highly reflective with little transmission to the bottom layer (near-field in Fig. 3d), the suppressive effect on the near-field is significantly less than in the case of a Au nanoparticle bilayer film. In the simulated spectra of Fig. 3c for Ag nanoparticles, a mild shoulder is observed at ~ 400 nm, which in the experimental spectra is hidden with only a mild inflection in that wavelength region. While the implication of this feature is not immediately clear at this point, some interesting implications in the near-field and detailed far-field (transmittance, absorptance and transmittance) spectra will be discussed in the later sections.

### Far-field optical properties in direct normal incidence

**Figure 4:**
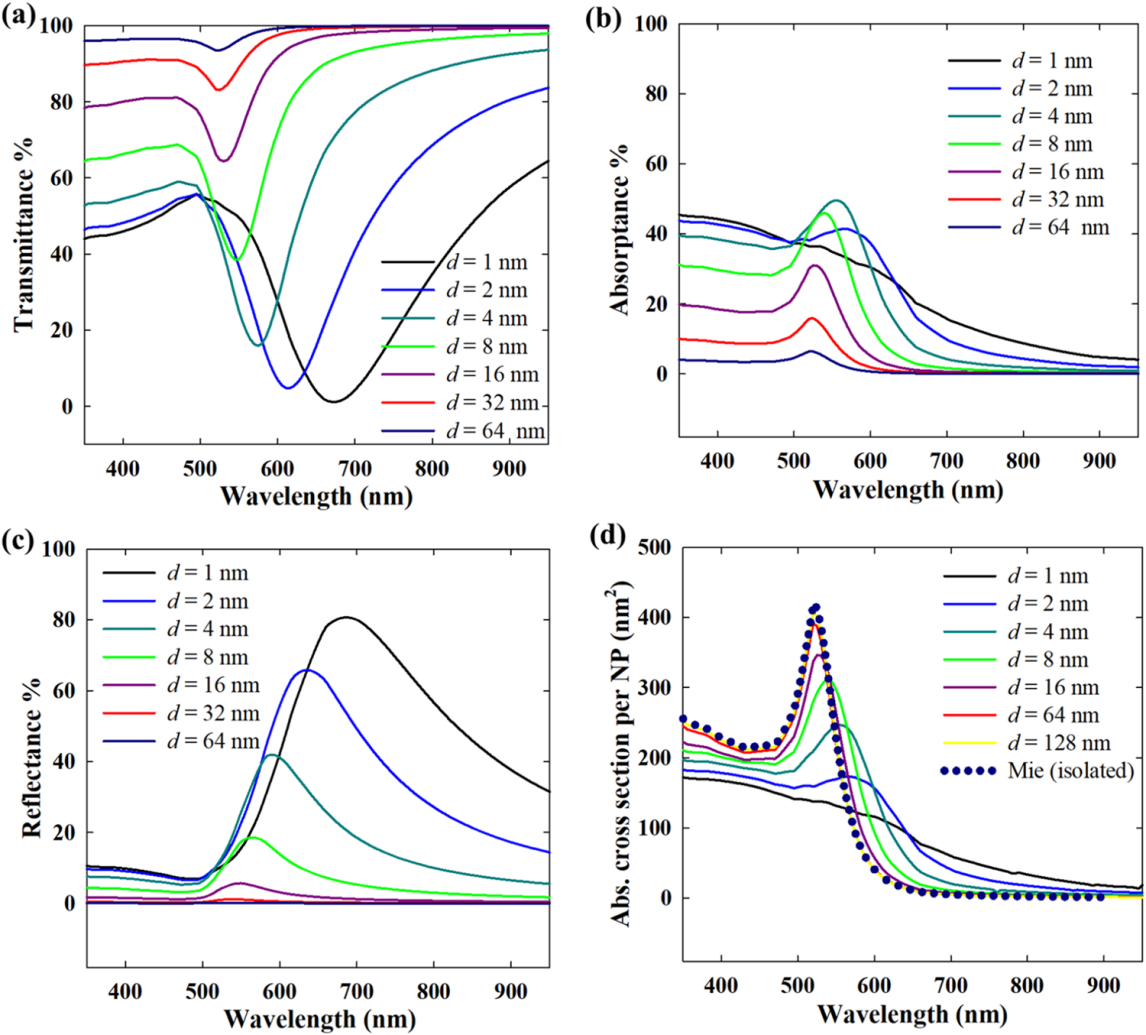
Effect of interparticle gap (*d*) in monolayer hexagonal close packed arrays of 20 nm Au nanoparticles in dielectric medium (*n* = 1.33): (**a**) transmittance, (**b**) absorptance, (**c**) reflectance, (**d**) per particle absorption cross section. The direction of incidence of illumination is normal in all cases.

**Figure 5:**
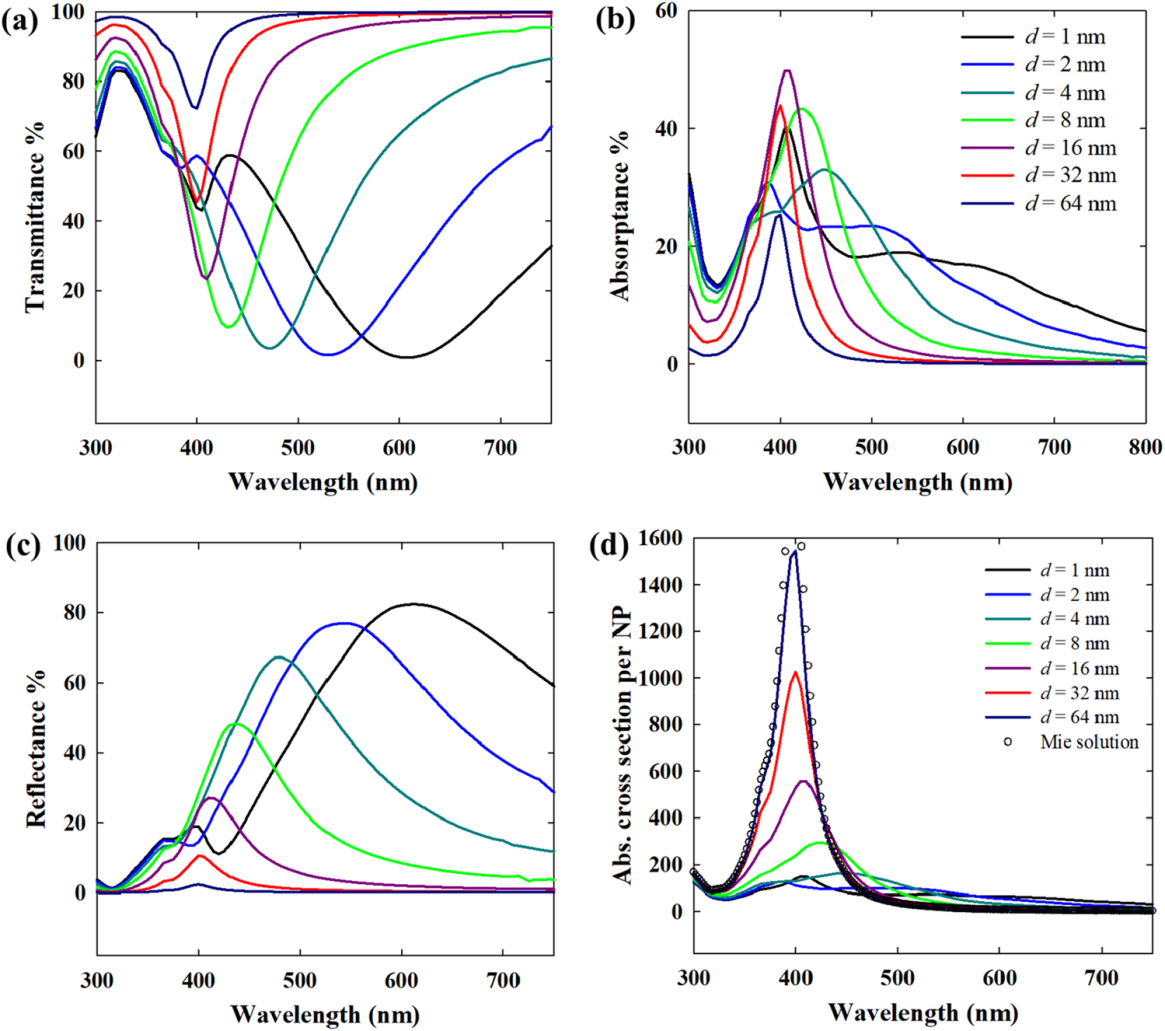
Effect of interparticle gap (*d*) in monolayer 2D hexagonal close packed arrays of 20 nm Ag nanoparticles in dielectric medium (*n* = 1.33): (**a**) transmittance, (**b**) absorptance, (**c**) reflectance, (**d**) per particle absorption cross section.

As the interparticle gap in a close packed nanoparticle array increases, the particle–particle coupling naturally weakens resulting in increasingly weaker lattice resonance. Also, gradually, the individual plasmon modes (predominantly dipolar) become more prominent as shown in Fig. 4 for Au and Fig. 5 for Ag nanoparticles. Thus, the plasmon band which is red-shifted due to the lattice resonance, now blue-shifts as the nanoparticles become distant from one another. At a small interparticle distance, the transmittance is naturally low (Figs. 4 and 5a), but it is mainly the reflectance that exerts the largest influence. Thus, the plasmonic enhancement by lattice resonance is not necessarily useful in applications where radiation/reflection losses are not desirable. For instance, in solar energy harvesting, a too dense packing would lead to the reflection of most of the incident energy, which is obviously unwanted in that case. For 20 nm Au nanoparticles in water, an interparticle gap of at least 32 nm is necessary to ensure minimum reflection losses while still maintaining considerable absorption for complete harvesting of the light in multiple layers. As shown in Figs. 4 and 5d, the individual absorption cross sections of the nanoparticles approach the Mie solution for isolated nanoparticles as the interparticle distance increases. The individual absorption cross section of 20 nm Au nanoparticles in a closely packed array is significantly lower with a weak plasmon band in contrast to isolated nanoparticles with only absorption and almost no scattering. Thus, the excitation of the plasmons in nanoparticles closely packed as an array leads to changes in the plasmon relaxation pathways with the radiative damping dominating over non-radiative damping. This has strong implications in plasmonic hot electron driven processes as the hot electrons typically result from non-radiative damping^34^.

The plasmonic response of Au nanoparticles under visible light is dampened by the interband transitions due to the low interband transition energy of Au (2.3 eV) that thus corresponds to this visible light wavelength range^35,36^. Since the interband transition in Ag requires a much larger energy (3.7 eV), with an overlap in the UV region, the plasmonic excitation in Ag nanoparticles under visible light is free from such direct interband transition losses. Thus, the plasmonic response of Ag nanoparticles is dominated by only free electron excitation in the visible range, hence, resulting in more efficient plasmonic excitation. In 20 nm diameter nanoparticles, while Au nanoparticles have almost no radiative damping i.e. scattering (1% < of total extinction), Ag nanoparticles have a small contribution (~ 5% of total extinction) from scattering at the localized plasmon resonance. This implies that, although 20 nm Ag nanoparticles in 2D arrays will exhibit lattice resonance mainly by near-field coupling, weak far-field coupling is also present due to the interaction of the scattered fields. As Ag nanoparticles individually have significantly higher optical cross sections than Au, relatively higher optical intensities are maintained for the same interparticle gap (Fig. 5a–c). With decreasing interparticle gap, the stronger plasmonic coupling is evident from the large bands in transmittance (dip) and reflectance (peak). Also, as shown in Fig. 5d, a greater distance as compared to Au nanoparticles is required for Ag nanoparticles to become completely decoupled. This is due to the far-field coupling of the Ag nanoparticles as will be explained later. Interestingly, if Fig. 5a,b are compared, the transmittance and absorption spectra for an interparticle gap = 1 nm or 2 nm show two distinct features. While the lowest transmittance dip is at 600 nm for the 1 nm interparticle gap case, the absorptance has the highest peak at around 400 nm with only a broad band at 600 nm. In the transmittance and reflectance, a small shoulder at ~ 400 nm is observed. Thus, the absorption maximum at 400 nm also indicates a strong plasmon resonance that relaxes mainly non-radiatively. For an interparticle gap of 2 nm, this peak in all three transmittance, absorptance and reflectance, is much weaker. While the experimental transmittance spectra for 20 nm nanoparticles with 2 nm interparticle gap in Fig. 3c do not show this shoulder clearly, the presence of a weak inflection can nonetheless be observed.

**Figure 6:**
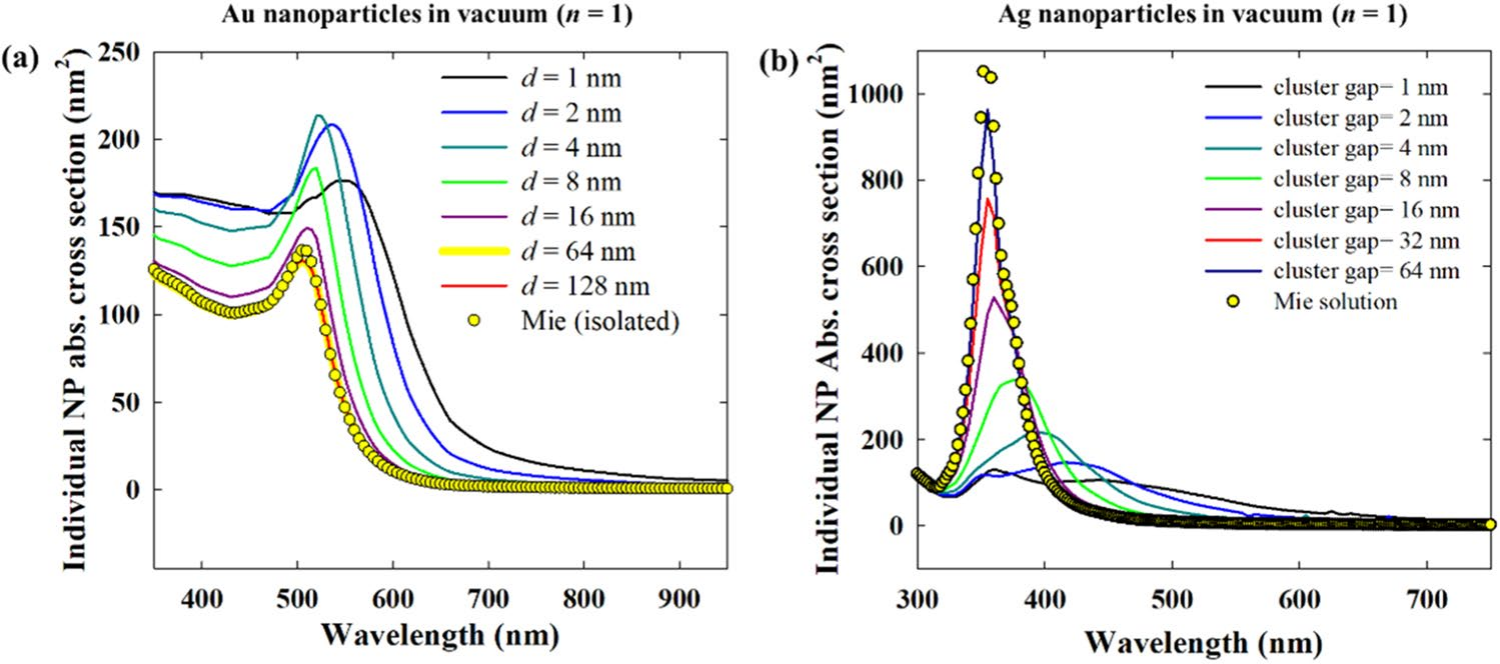
Effect of interparticle gap (*d*) in monolayer 2D hexagonal close packed arrays of 20 nm Ag nanoparticles in vacuum (*n* = 1), comparing the absorption cross section per particle.

In contrast to a dielectric water medium (*n* = 1.33) as shown in Figs. 4 and 5, in vacuum, the interparticle coupling is suppressed due to the absence of a dielectric environment. When the surrounding medium is a dielectric, it also polarizes with the plasmonic nanoparticles in response to the incident field, thereby enhancing the interparticle coupling^37,38^. Thus, in vacuum (*n* = 1), it takes just a short interparticle distance for the nanoparticles to become isolated from interparticle interactions (Fig. 6). Also, the individual nanoparticle absorption cross section varies differently for Au and Ag. For small interparticle gaps (i.e. strong plasmonic coupling), the individual absorption intensity of 20 nm Au nanoparticles is higher than in the isolated nanoparticles (Fig. 6a). This implies a stronger contribution of non-radiative damping of the excited plasmons as well as interband transitions resulting in the absorption, when the nanoparticles are in vacuum (or gaseous medium). Thus, in gas phase applications of Au nanoparticles, strong interparticle coupling can enhance photothermal processes. In contrast, strongly coupled Ag nanoparticles have significantly lower absorption per particle than in the case when they are isolated (Fig. 6b), as the radiative damping of the excited plasmons becomes more dominant.

Another interesting behavior is observed in the transition when going from clusters to infinite films. As one goes from a single nanoparticle to 2D finite arrays (or clusters) of increasing size, the optical properties vary strongly^2^. The unit cell approximation is only valid for cases where the film is infinitely larger than the wavelength of incidence. However, even quite large clusters of hundreds of nanoparticles exist where this condition is not applicable. Zakomirnyi et al. showed that even larger arrays, up to 100 × 100 nanoparticles, have optical responses that are significantly different from the ones calculated for infinite arrays with the same nanoparticle sizes and interparticle distance^39^. While this aspect is not discussed in detail in the present work, further investigation of clusters of intermediate sizes remains interesting for future studies.

### Near-field optical properties in direct normal incidence

Strong amplification of the evanescent electric field close to the resonating plasmonic nanoparticles is a characteristic optical effect of plasmonic nanoparticles. In this section, the near-field profiles with varying interparticle gap are discussed first, followed by the near-field spectra. In normal incidence i.e. *θ* = 0°, and the two perpendicular modes of polarization *φ* = 0° and *φ* = 90°, according to Fig. 1c, are also discussed as the near-field properties depend on the angle of polarization, while far-field properties exhibit rotational symmetry.

**Figure 7:**
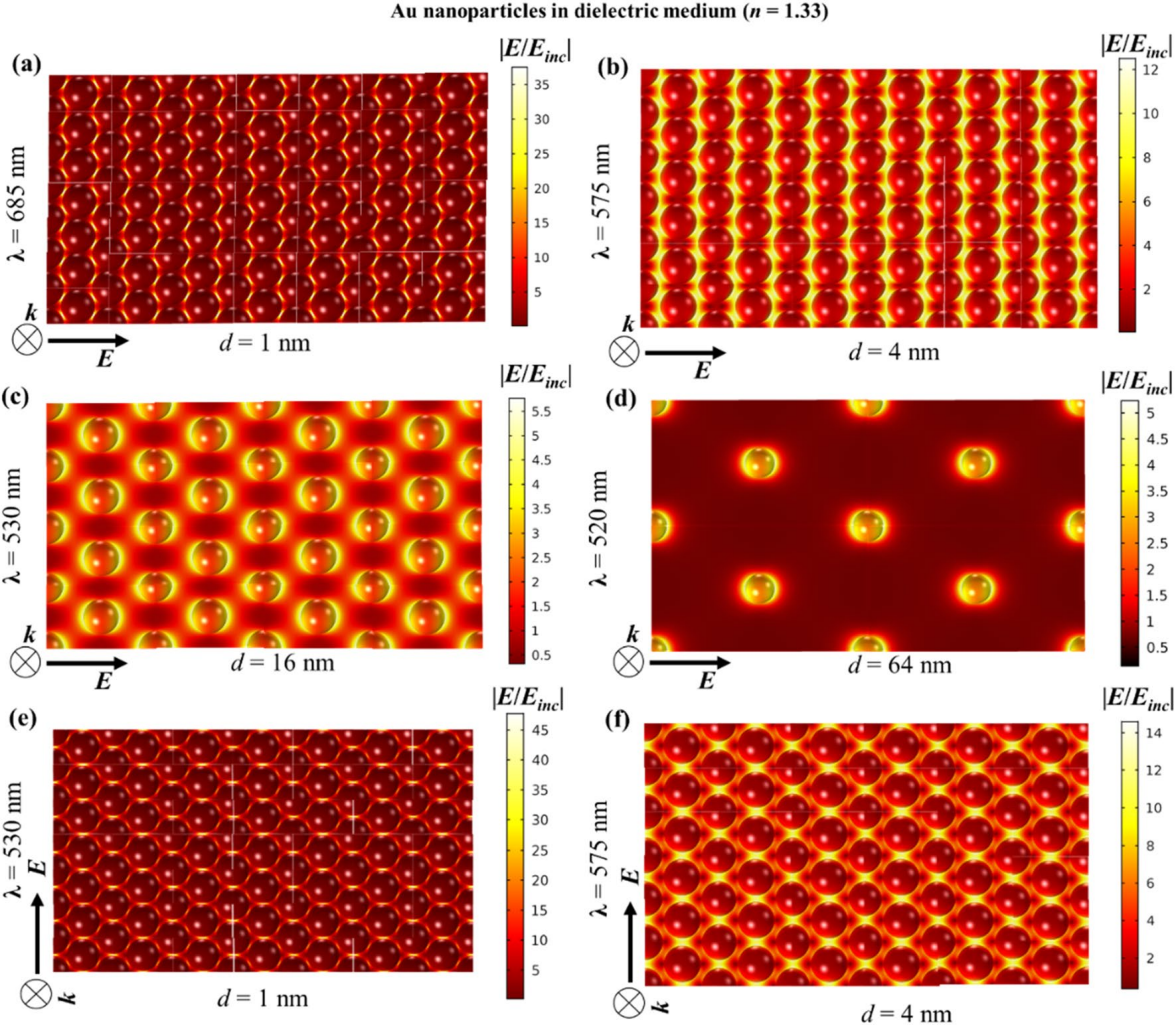
Near-field enhancement w.r.t*.* the incident field for 2D hexagonal monolayer array of 20 nm Au nanoparticle in dielectric medium (*n* = 1.33) for different interparticle gaps, *d*: (**a**) 1 nm, (**b**) 4 nm, (**c**) 16 nm and (**d**) 64 nm. (**e**) and (**f**) Shows the near-field profiles at vertical polarization for interparticle gaps 1 and 4 nm respectively. At normal incidence i.e. *θ* = 0°, (**a**–**d**) are for horizontal polarization i.e. *φ* = 0° and vertical polarization i.e. *φ* = 90°.

**Figure 8:**
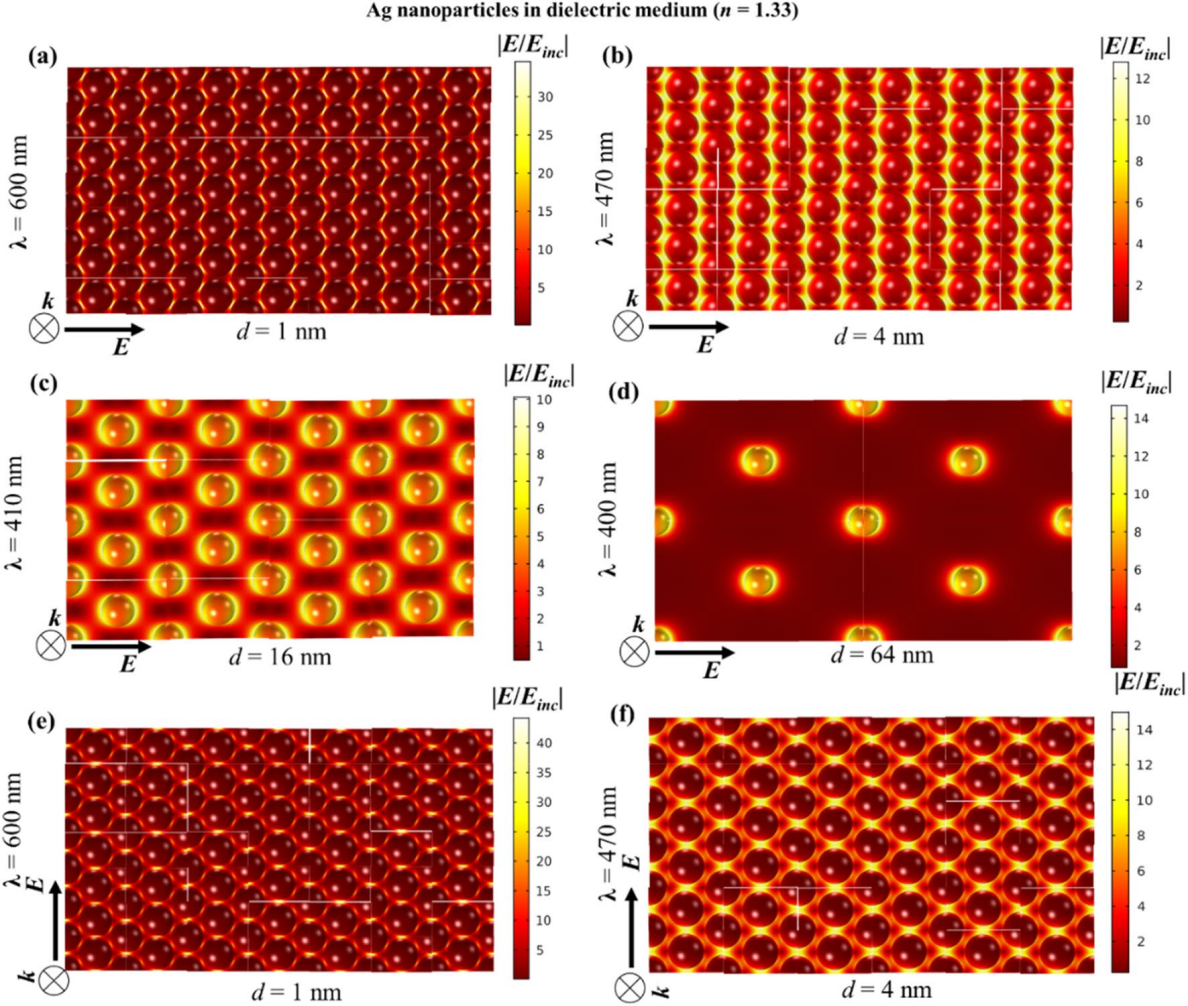
Near-field enhancement w.r.t. the incident field for 2D hexagonal monolayer array of 20 nm Ag nanoparticle in dielectric medium (*n* = 1.33) for different interparticle gaps, *d*: (**a**) 1 nm, (**b**) 4 nm, (**c**) 16 nm and (**d**) 64 nm. (**e**,**f**) Shows the near-field profiles at vertical polarization for interparticle gaps 1 and 4 nm respectively. At normal incidence i.e. *θ* = 0°, (**a**–**d**) are for horizontal polarization i.e. *φ* = 0° and vertical polarization i.e. *φ* = 90°.

This near-field enhancement is much stronger for isolated Ag nanoparticles than for isolated Au nanoparticles, as plasmons are significantly dampened by interband transitions in Au^40^. In any case, it is known that the plasmonic coupling between adjacent nanostructures results in even stronger near-field enhancement in the interparticle gap^41^. In plasmonic nanoparticle arrays, the lattice resonance by near-field interactions also results in extremely high near-field enhancement relative to isolated nanoparticles (Figs. 7 and 8). The maximum intensity of the near-field enhancement increases with decreasing interparticle gap. However, at the same time, the enhanced near-field, which is a non-propagating standing wave, also gets confined to a smaller space as the interparticle gap shortens. Such high enhancement into small space is, for instance, promising for single molecule detection by signal enhancement^42^. The near-field enhancement also indicates the strength of plasmonic coupling with increasing interparticle distance. At 64 nm interparticle spacing, the 20 nm Au nanoparticles become completely decoupled from each other with near-field enhancement as strong as that for individual nanoparticle (Fig. 7 and Fig. S5a).

Although individual Ag nanoparticles have stronger near-field enhancement than Au nanoparticles, in a close packed array, this advantage vanishes. As shown in Fig. 8a,b, the maximum intensity of the near-field enhancement in the Ag nanoparticle array is of the same order as that of Au nanoparticle arrays in Fig. 7a,b. However, as the interparticle distance increases, the difference between both cases increases and Ag nanoparticles tend to show significantly higher field enhancement. At an interparticle distance of 64 nm, Ag nanoparticles also have near-field properties similar to individual particles (Fig. S5b). In Figs. 7 and 8, at an interparticle gap of 16 nm, there is a significant difference in the near-field enhancement of Au and Ag. As the nanoparticles are sufficiently distant from one another, the near-field enhancement of Ag is much larger than that of Au, like in the case of isolated nanoparticles. This means that for a 16 nm interparticle gap, the surface can be efficiently covered by Ag nanoparticles displaying higher near-field enhancement compared to Au. Now, when moving towards denser surface coverages, the advantage of the stronger near-field enhancement of Ag with respect to Au diminishes.

Rahmani et al. showed for a plasmonic oligomer that the far-field spectra (transmittance, reflectance and absorptance) are not influenced by the angle of polarization at normal incidence, while the near-field characteristics do change with polarization^43^. It is shown that since the near-field profiles are determined by the particular mode superpositions excited by the incident field, they vary with the polarization. In contrast, due to the rotational symmetry in the oligomers, the far-field properties are left unaffected by the polarization. A similar trend can also be seen in Figs. 7 and 8 for Au and Ag nanoparticles respectively, where the horizontal and vertical (w.r.t. the page layout) polarization give rise to starkly different near-field profiles with different maximum values. The consequence of this can be interesting in surface enhanced spectroscopy applications of self-assembled nanoparticle films.

**Figure 9:**
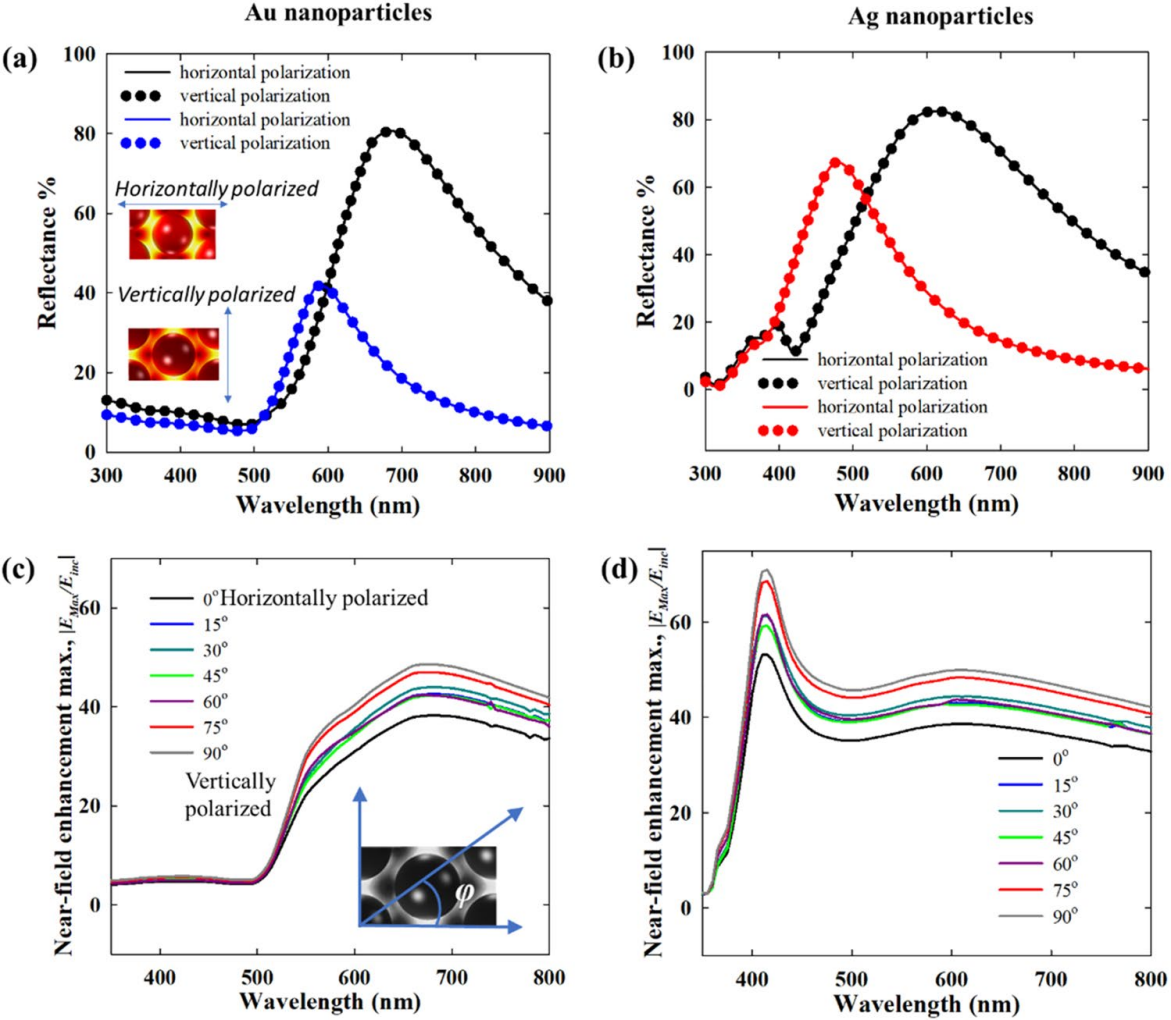
Comparison of reflectance spectra of Au (**a**) and Ag (**b**) nanoparticle assemblies under normal illumination with 1 nm and 4 nm interparticle gaps at two perpendicular polarizations. The maximum near-field enhancement spectra of Au (**c**) and Ag (**d**) nanoparticle assemblies (shown only for an interparticle gap of 1 nm) at different polarization angles denoted by *φ*. Horizontal polarization, *φ* = 0° and vertical polarization, *φ* = 90° are defined w.r.t to the plane of the screen. The incident wave is normal to the plane of the nanoparticle film in all cases. (embedding medium, *n* = 1.33).

While the near-field profiles are different for two perpendicular directions of polarization, the far-field response is polarization independent. This is obvious from Fig. 9a,b, where the reflectance spectra for vertical and horizontal polarization merge together for both Au and Ag nanoparticles. It could already be seen in Figs. 7 and 8 that the near-field profiles and the range are both different for the two directions of polarization. The near-field maximum spectra in Fig. 9c,d show the complete trend over a broad wavelength range. Clearly, the near-field maximum for the vertical polarization is higher than for all other polarization angles defined by *φ* in the plane cutting through the center of the nanoparticles along the plane of the film. The near-field enhancements averaged over the cut-plane for horizontal and vertical polarization are, however, much closer as the near-field profiles are only differently distributed with respect to the incident polarization in the two cases and the average effect is expectedly low (Fig. S6). Figure 9c,d also show that for Au, the high near-field enhancement due to lattice resonance is observed only at wavelengths over 500 nm. However, for Ag, a peak in the near-field spectra is observed close to 400 nm. As discussed above, this resonance mode is observed in the far-field spectra also in the form a shoulder in transmittance and reflectance. While in the absorptance, a strong global maximum is observed in that region. Thus, it is interesting that an overall strong near-field enhancement is obtained over the entire visible range in the case of close packed Ag nanoparticle assemblies.

### Effect of substrate in direct normal incidence

**Figure 10:**
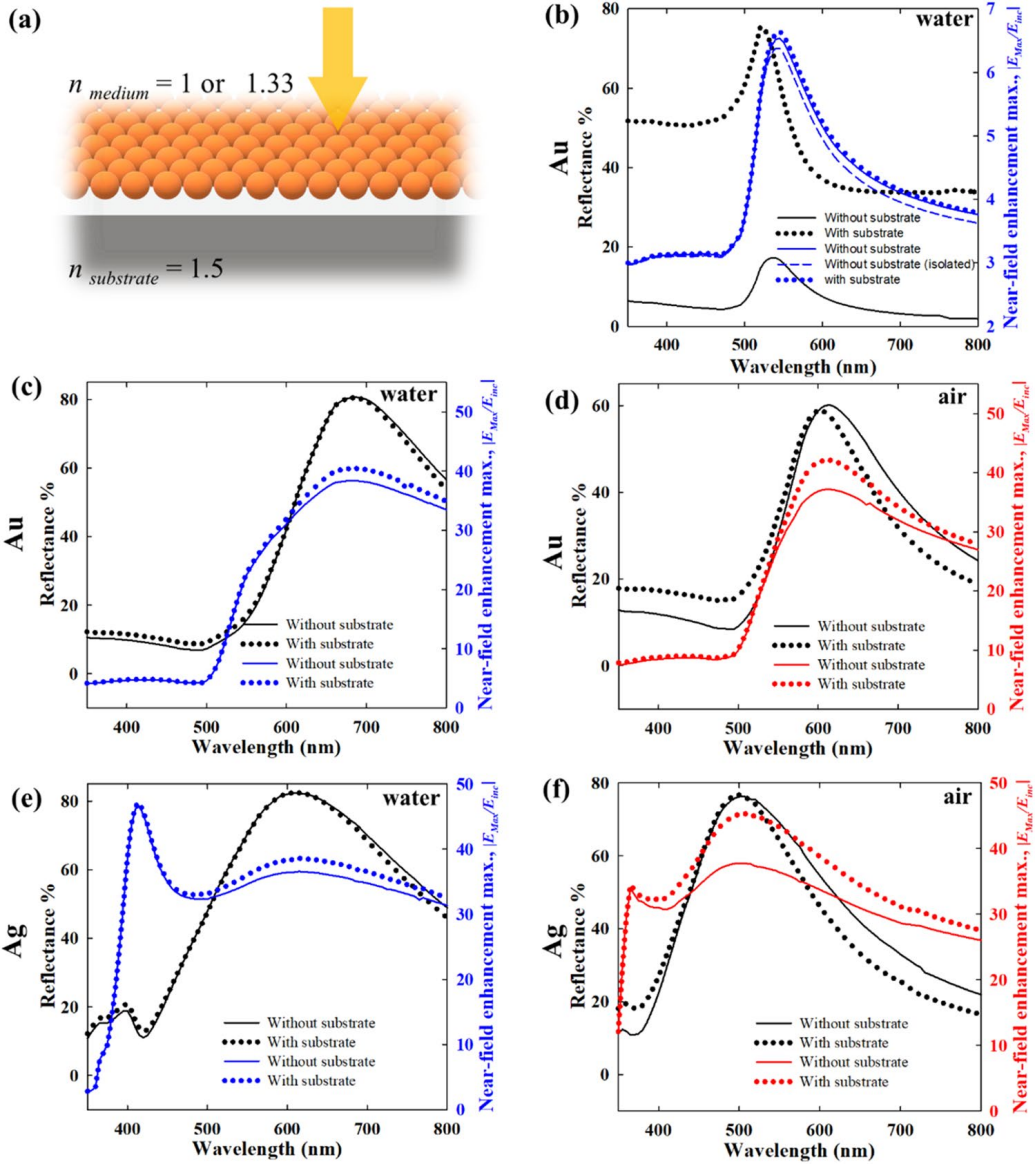
(**a**) Schematic of nanoparticles on substrate with normal direct incidence i.e. *θ* = 0°. (**b**) Effect of substrate (*n*_*sub*_ = 1.5, *n*_*medium*_ = 1.33) on reflectance and near-field enhancement maximum spectra for Au nanoparticle assembly with large interparticle distance, 64 nm. Effect of substrate for (**c**) *n*_*medium*_ = 1.33 and (**d**) *n*_*medium*_ = 1 with close packed Au nanoparticle assembly i.e. interparticle distance 1 nm, comparing reflectance and near-field enhancement maximum spectra. Effect of substrate for (**e**) *n*_*medium*_ = 1.33 and (**f**) *n*_*medium*_ = 1 with close packed Ag nanoparticle assembly i.e. interparticle distance 1 nm, comparing reflectance and near-field enhancement maximum spectra.

While the discussion so far involved only the nanoparticle arrays/films, in real applications the substrate onto which the films are immobilized, also plays an important role. Figure 10 elucidates the effect of a substrate in terms of reflectance and near-field maximum spectra by introducing a dielectric substrate of *n* = 1.5 (Fig. 10a), a refractive index value typical for glassy materials. The effect of the dielectric substrate is mainly manifested in the reflectance. In the presence of the nanoparticles, the wave transmitted through the nanoparticle array undergoes reflection and transmission. This reflected wave again interacts with the nanoparticles to give rise to a net effect. Thus, it is expected that as the interparticle gap decreases, i.e. the transmitted wave weakens, the effect of the substrate also decreases.

As shown in Fig. 10b, the effect of substrate for an isolated Au nanoparticle, i.e. interparticle distance 64 nm, is quite strong when the embedding medium is a dielectric. With substrate, the reflectance is shifted by a significant margin from that without substrate, however, without any significant change in the spectral features. On the other hand, the near-field maximum spectra show very small differences between the with-and-without substrate cases. This corroborates the point that the effect of the substrate is majorly to enhance the reflectance, which weakly influences the plasmonic response of the nanoparticles. However, for close packed films as shown in Fig. 10c,d, irrespective of the embedding medium being air or water, the effect of the substrate remains quite small. A similar trend is also observed for Ag nanoparticles as shown in Fig. 10e,f.

### Far-field and near-field optical properties in the Kretschmann (ATR) configuration

**Figure 11:**
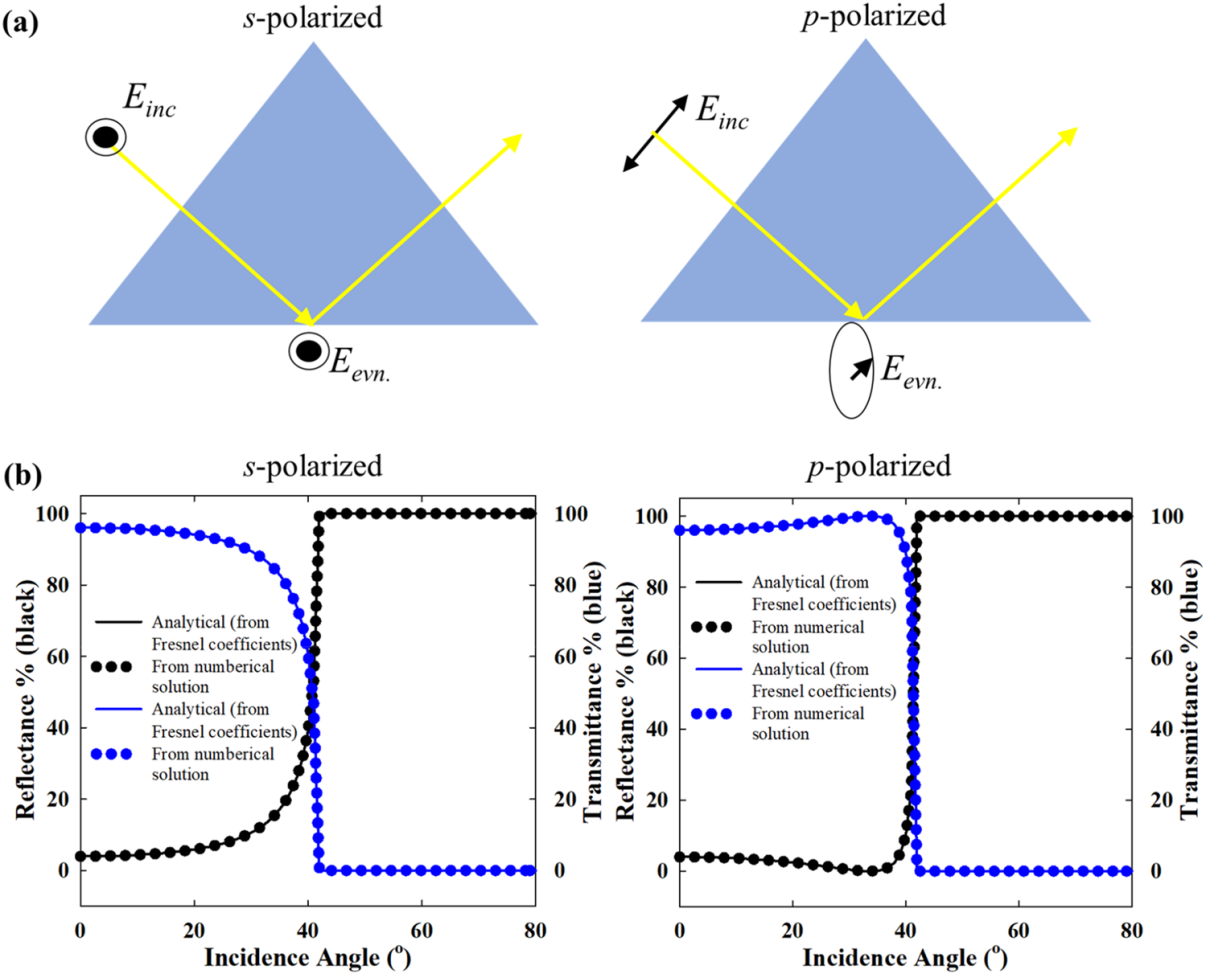
(**a**) Schematic representation of the evanescent field polarization for *s*- and *p*-polarized incidence in the Kretschmann configuration. (**b**) Validation of the angle resolved electromagnetic models of the Kretschmann configuration (without nanoparticles) by comparison with optical spectra obtained from the Fresnel coefficients for *p-*polarized i.e. TM polarized and *s*-polarized i.e. TE polarized incidence.

In order to excite the surface plasmons in a planar thin film, it is necessary to implement specific geometric arrangements such as the Kretschmann (ATR) configuration to compensate for the momentum mismatch between the incident photon and the plasmon wave^44^. In contrast to thin planar solid Au films, for a nanostructure array like a self-assembled nanoparticle film, the lattice resonance can be excited by direct illumination from a vacuum or dielectric medium. However, it is interesting to investigate the excitation of the lattice plasmons in the Kretschmann configuration, as many sensing applications utilize this arrangement with completely different underlying optics. Firstly, when the incident beam undergoes total internal reflection in the Kretschmann configuration, the plasmonic excitation of the nanoparticle film is only possible by the standing evanescent field on the other side of the prism coupler (Fig. 1d). As there is no transmission possible, the reflected intensity is only due to the absorption by the film due to the plasmonic excitation. Importantly, the evanescent field can interact with the nanoparticles only up to a short distance from the prism-dielectric interface as the field intensity decays exponentially with distance. Also, the evanescent fields due to *s* and *p*-polarized (TE and TM polarized) light have totally different directionality, as shown in Fig. 11a^45^. For *s*-polarization, the evanescent field is linearly polarized, perpendicular to the plane of incidence. However, the evanescent field for *p*-polarized incidence is elliptically polarized in the plane of incidence that “cartwheels” along the boundary^46^. Also, the evanescent field intensity for *p*-polarized incidence is significantly larger than for *s*-polarized incidence. Due to these differences, it is expected that the optical spectra for the two directions of polarization are significantly different.

In ATR-FTIR spectroscopy, it has been shown that while the *p*-polarized light can probe the vibration modes parallel and perpendicular to the sample film, the *s*-polarized beam probes solely the components of the vibrational modes parallel within the plane of the sample film^47^. The accuracy of the angle-resolved electromagnetic models was ascertained by comparison with analytical spectra from Fresnel coefficients. As shown in Fig. 11b, the computed spectra for *p* and *s*-polarized incidence in the ATR configuration without nanoparticles match perfectly with the analytical solution. The nanoparticles were introduced in the same numerical models for further calculations. From a computational point of view, obtaining solutions for the direct incidence at different angles poses significant difficulties due to the Rayleigh Wood’s anomalies^48,49^. These surface wave anomalies occur at certain wavelengths due to the diffracted waves that arises due to the periodicity of the metallic structures, and propagate tangentially to the surface of the nanostructure array^50^. The tangential wave propagation poses difficulties with the periodic boundary conditions on the side walls of the unit-cell domain, whereas for the Kretschmann (ATR) configuration, such difficulties were not encountered in the numerical solution. The consistency in the computed spectra in the following discussion further corroborates this point.

**Figure 12:**
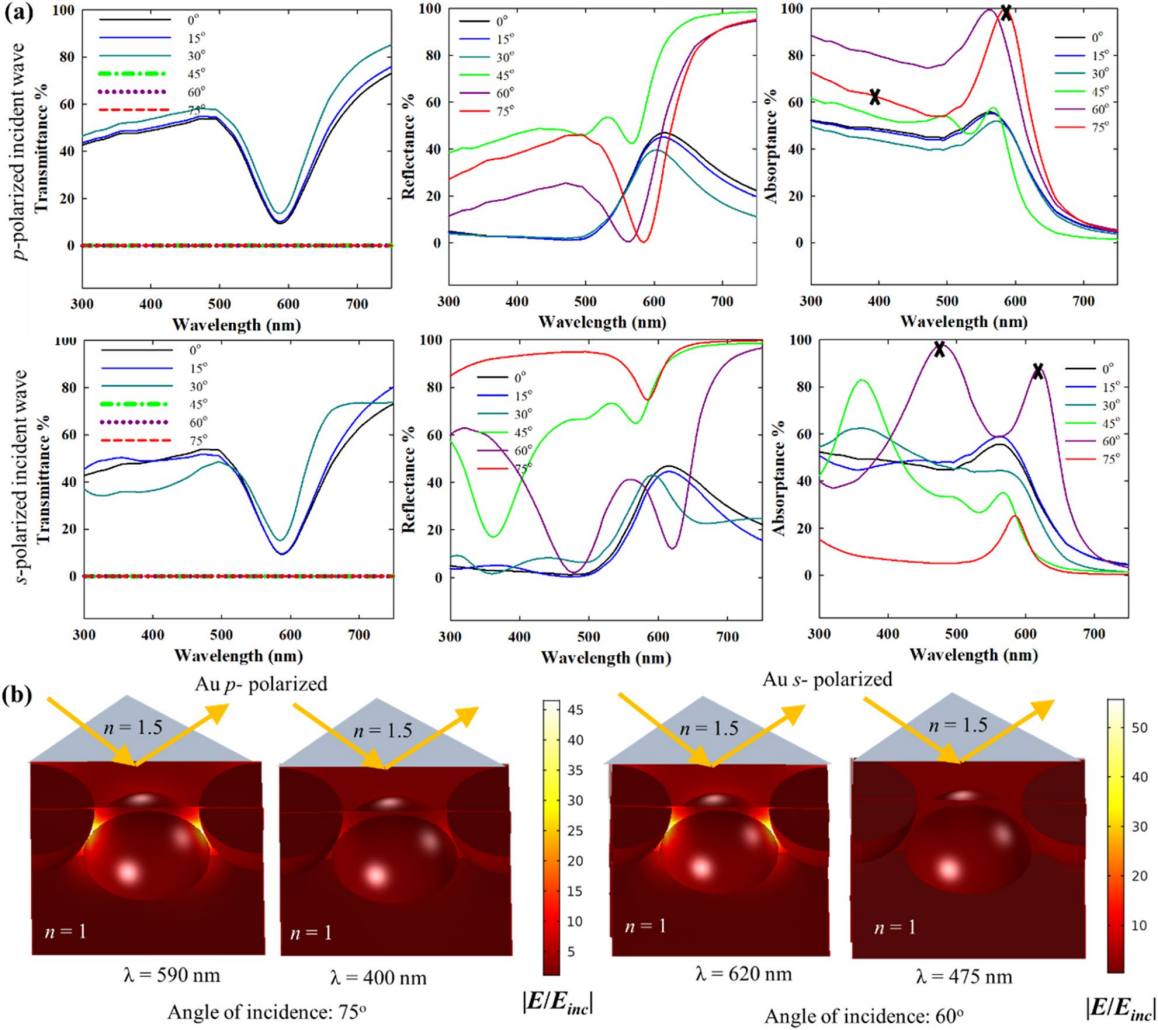
(**a**) Transmittance, reflectance and absorptance spectra of a close packed 20 nm Au nanoparticle film in the Kretschmann i.e. ATR configuration for *p*- and *s*-polarized incident wave, and varying incident angle (*n* = 1.5 for the denser medium, *n* = 1 for the lighter medium and interparticle gap: 1 nm). (**b**) Near-field enhancement with respect to the background evanescent field (in absence of the nanoparticles) for *p* and *s*-polarized (or TM and TE polarized) incidence.

**Figure 13:**
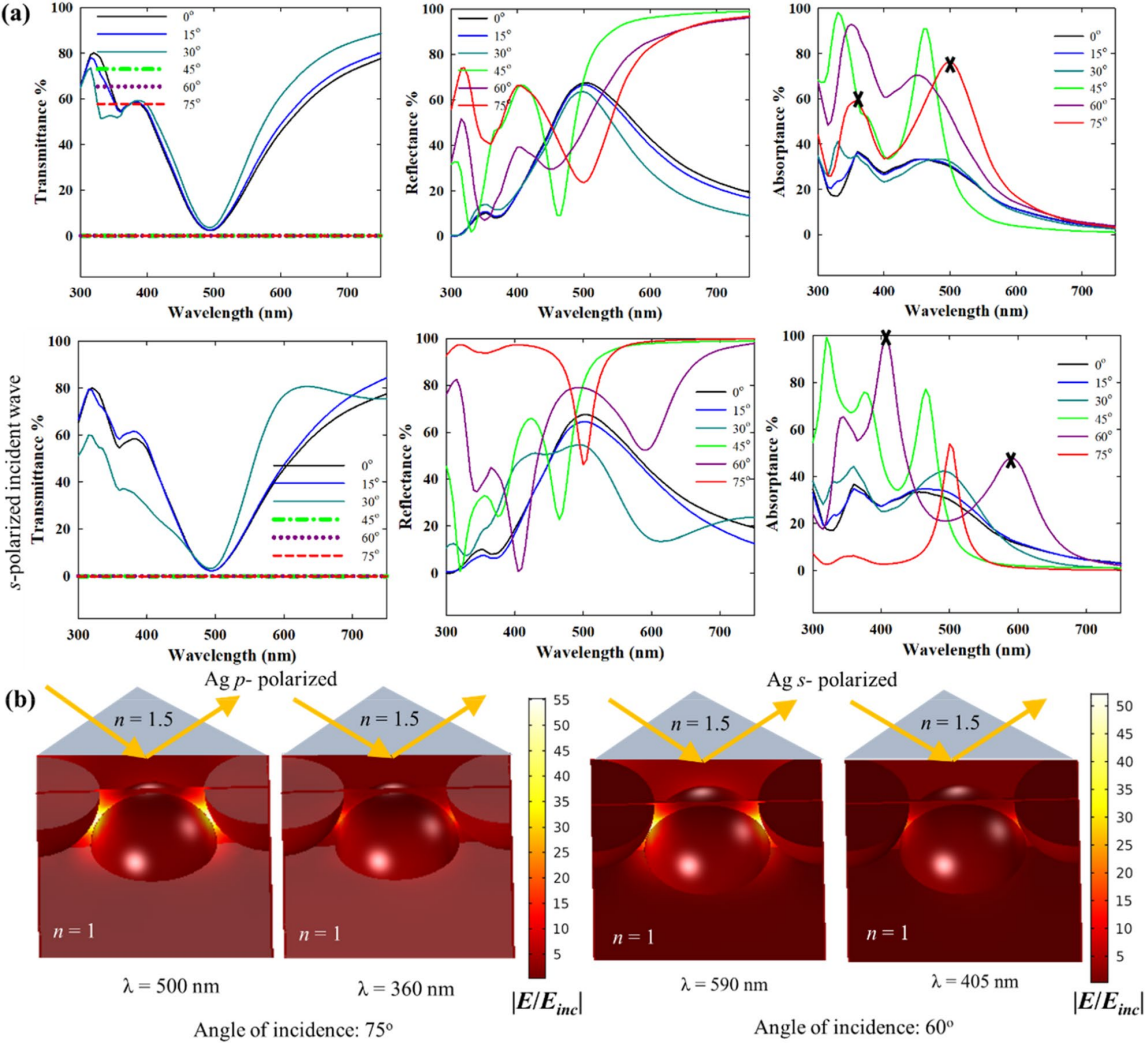
Transmittance, reflectance and absorptance spectra of a close packed 20 nm Ag nanoparticle film in the Kretschmann i.e. ATR configuration for *p*- and *s*-polarized incident wave, and varying incident angle (*n* = 1.5 for the denser medium, *n* = 1 for the lighter medium and interparticle gap: 1 nm). (**b**) Near-field enhancement with respect to the background evanescent field (in absence of the nanoparticles) for *p-* and *s*-polarized (or TM and TE polarized) incidence.

Figures 12 and 13 compare the optical spectra of close packed Au and Ag nanoparticle monolayer films in the Kretschmann configuration for both *p*- and *s*- polarized incident beams at different incident angles. For the combination of a glass prism (*n* = 1.5) and air (*n* = 1) the critical angle of incidence is 41.8°. Thus, for incident angles above 41.8°, the transmittance becomes zero and the incident power goes only to absorptance and reflectance (Figs. 12a and 13a). Up to 30°, the transmittance, reflectance and absorptance spectra remain qualitatively similar to the spectra for normal direct incidence as the refracted wave that interacts with the nanoparticles has similar properties as the incident wave. At 45° incidence and onwards, the lattice plasmons are excited by the evanescent wave. The lattice resonance of the nanoparticle array excited by the evanescent wave is marked by a dip in reflectance in agreement with Manera et al.^51^, which is red-shifted from individual LSPR of the nanoparticles (in air).

The differences in the evanescent fields from *p*- and *s*-polarized incidence have been discussed above in connection to Fig. 11b. These differences, along with the dependence of the evanescent field intensity also on the angle of incidence and wavelength of incidence, lead to the variabilities in the spectra in Figs. 12 and 13 for cases beyond the critical angle. As shown in Fig. 11b, the evanescent field due to the *p*-polarized incidence consists of two components: one is in the plane of the nanoparticle film and the other one is out of the plane. The in-plane component dominates the optical response due to the interparticle coupling resulting in enhanced light-nanoparticle interactions. This explains the near-field enhancement plots in Figs. 12b and 13b for *p*-polarized incidence that shows primarily an enhancement in the interparticle gaps.

**Figure 14:**
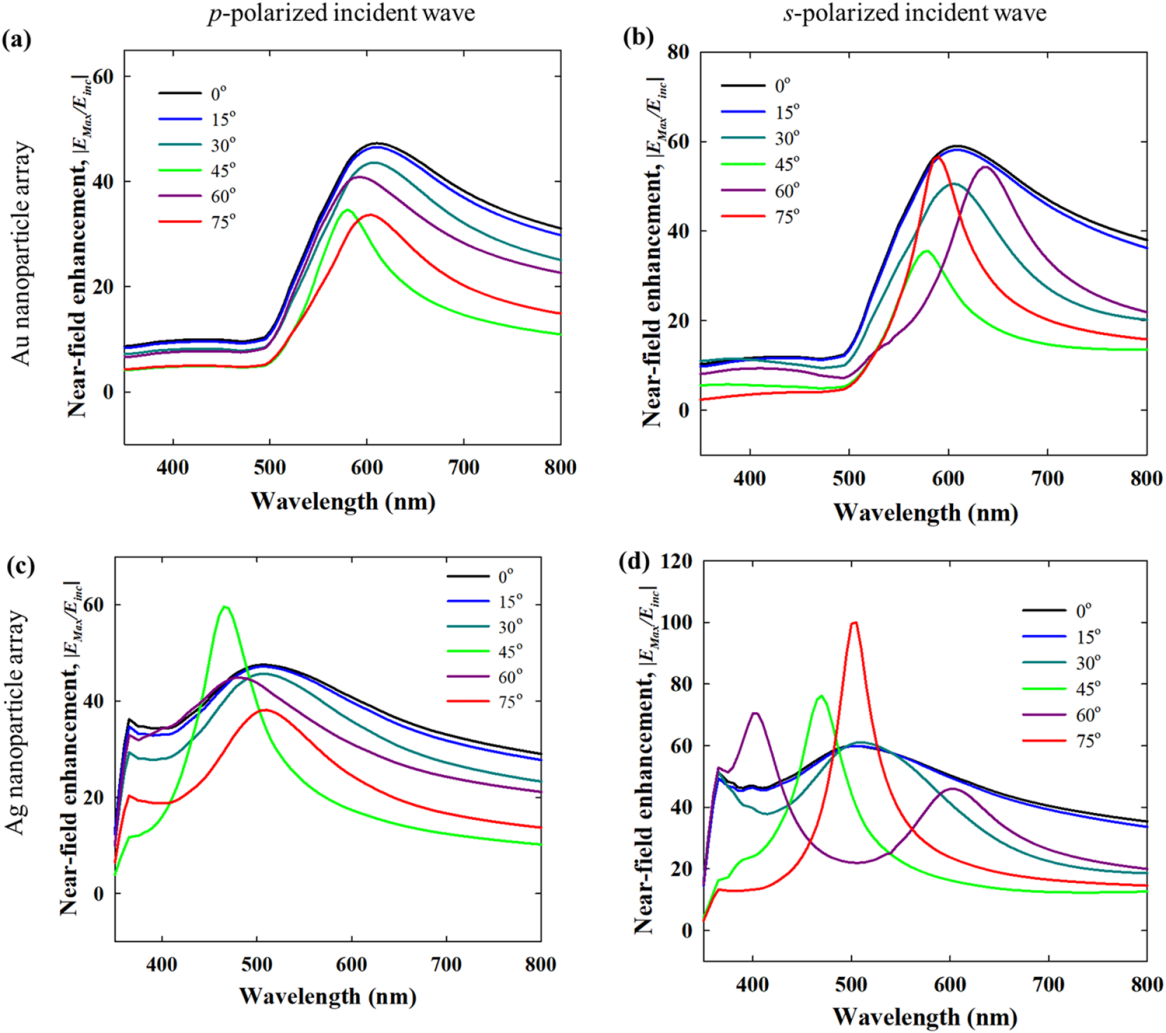
Near-field maximum spectra of close packed 20 nm Au (**a**,**b**) and Ag (**c**,**d**) nanoparticle films in the Kretschmann i.e. ATR configuration for *p*- and *s*-polarized (or TM and TE polarized) incident wave, and varying incident angle (*n* = 1.5 for the denser medium, *n* = 1 for the lighter medium and interparticle gap: 1 nm). Legend: for each color from black to red, the solid lines represent near-field enhancement w.r.t. the incident field (without the nanoparticles and the Kretschmann configuration).

The *p*- and *s*-polarized spectra in the Kretschmann configuration have qualitative similarities with the *p*- and *s*-polarised spectra of plasmonic nanostructure arrays under direct angular incidence^52^. At *p*-polarization, the Au nanoparticle film has high broadband absorption for larger incidence angles, > 60°. The strong and broadband absorptance can be interesting for light harvesting applications. As shown in Fig. 4 for a close packed nanoparticle array (i.e. interparticle distance = 1 nm) in direct normal incidence, the strong radiative damping of the plasmons excited at lattice resonance results in strong reflection and low absorptance. In contrast, in the Kretschmann configuration, the damping pathway is now switched to non-radiative damping, which is again advantageous for light harvesting applications. Apparently, the interplay of factors determining the local intensity of evanescent field is more complex for *s*-polarized incidence, as indicated by the multiple features in the reflectance spectra for 45°, 60° and 75° incidence angles. However, in Fig. 14b, the near-field maximum spectra show a well resolved single band indicating the position of the plasmon resonance, in all these cases. This helps in the elucidation of the plasmonic response as near-field enhancement is the “signature” of the plasmon resonance. In Fig. 12b, the near-field enhancement for *p-*polarized incidence at 590 nm is due to the plasmon resonance, while at 400 nm, the near-field enhancement is significantly lower due to interband transitions. For *s-*polarization, the low near field enhancement at 475 nm despite the existence of a band in the optical spectra indicates that its origin is not from plasmon resonance. On the other hand, at 620 nm, the strong near-field enhancement indicates the plasmon resonance, in line with the near-field spectra in Fig. 14b. It is important to note that the near-field enhancement in Figs. 12 and 13 is shown with respect to the resulting background evanescent field. The background evanescent electric field is weaker in the *s*-polarized case due to faster attenuation.

Although there are some similarities in the optical responses of Au and Ag nanoparticles in Figs. 12 and 13 when the same Kretschmann configuration is used, the optical spectra of Ag overall are significantly different from those of Au due the differences in intrinsic optical properties. The variability of the local evanescent field intensity with polarization, incidence angle and wavelength results in multiple features. The absence of interband transitions in the visible range for Ag (while present for Au) alters the lattice resonance conditions as well as the overall optical properties. Up to 30° incidence angle in Fig. 13a, the optical spectra are similar to direct normal incidence, and for 45° (the critical angle of incidence) and beyond, the optical spectra result from the plasmonic excitation by the evanescent wave. Interestingly, for Ag multiple plasmon resonance modes can be located in the spectra which is confirmed by the near-field maximum spectra in Fig. 14. For example, in the case of 60° *s*-polarized incidence in Fig. 13a, the two bands at ~ 600 and ~ 400 nm coincide with the two bands in the near-field maximum spectra in Fig. 14. Similarly, for *p*-polarized incidence, the two bands at ~ 500 and ~ 360 nm for 45°, 60° and 75° incidence angles can be traced also in the near-field spectra in Fig. 14 indicating different resonant modes. For the *s*-polarized case, similar features are expressed with a weaker intensity. Interestingly, this feature is also present for incidence angles below the critical angle when there is no total internal reflection.

For both Ag and Au nanoparticles, the reflectance and absorptance spectra in the Krestchmann configuration are determined by the local variability of the evanescent field with polarization, angle of incidence and wavelength. While the optical far-field spectra are determined by a lot of influencing factors, the near-field spectra with respect to the background field (in absence of the nanoparticles) can show the position of the plasmon band unambiguously. Some of the features in Fig. 14 have already been discussed in connection to the intensity spectra above. Generally, the near-field maximum spectra in Fig. 14 define the plasmon bands with more clarity. As shown above, the coincidence of the bands in the intensity (reflectance or absorptance) spectra and near-field spectra confirms the position of the plasmon resonance. Regarding the extent of the near-field enhancement, the Kretschmann configuration yields comparable or in some cases even stronger near-field enhancement than direct incidence. For instance, with *s*-polarized incidence at 75° incidence angle, the near-field enhancement for Ag nanoparticles is significantly stronger than at 0°. For Ag nanoparticles and at 45° incidence angle, both *p*- and *s*-polarization seem to yield stronger near-field enhancement as compared to direct incidence. As the near-field enhancement in Fig. 14 is shown with respect to the incident field, it is also useful to evaluate the enhancement with respect to the background evanescent field in absence of the nanoparticles. As shown in Fig. S7, the near-field enhancement with respect to the background evanescent field can be very high for larger incident angles (e.g. 60° or 75°). While this is not an enhancement over the incident field, such large enhancement over the background evanescent field can also be interesting in situations where the ATR configuration has to be used with a large angle of incidence. Experimental verification of these results is an interesting future endeavour with promising implications in surface enhanced spectroscopy applications.

**Figure 15:**
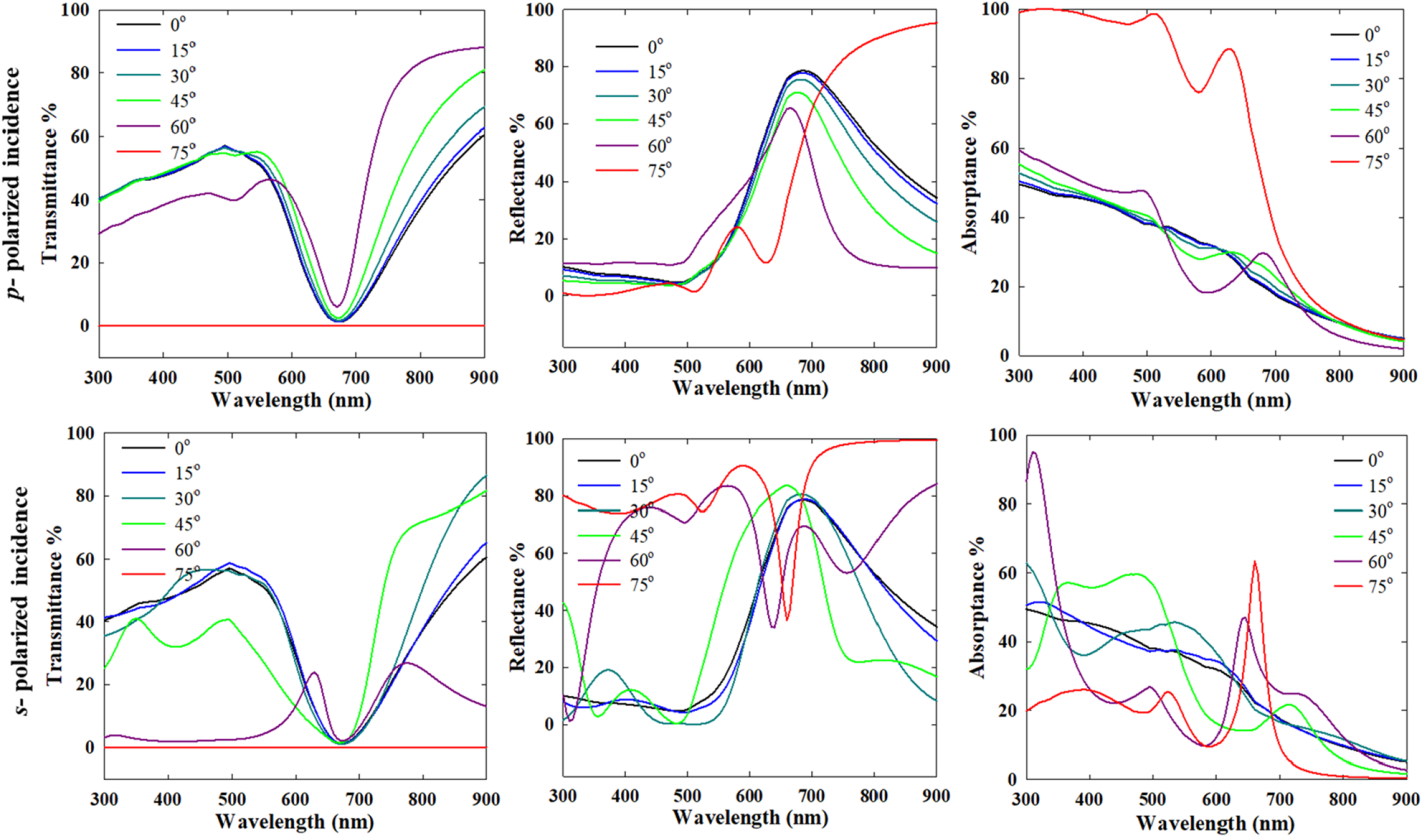
Transmittance, reflectance and absorptance spectra of a close packed 20 nm Au nanoparticle film in the Kretschmann i.e. ATR configuration for *p*-polarized and *s*-polarized incident wave, and varying incident angle (*n* = 1.5 for the denser medium, *n* = 1.33 for the lighter medium).

A final important aspect is the extrapolation of the results in the Kretschmann configuration to different dielectric media, packing configuration and so on. First, a dielectric medium with a higher refractive index will push the critical angle to a larger value. For example, for a dielectric medium of *n* = 1.33 and a prism coupler of *n* = 1.5, the critical angle becomes 62.6°. Thus, unlike Figs. 12 and 13, the zero transmittance spectrum is now only obtained at 75° incidence angle for the cases shown in Fig. 15. Similarly, the reflectance and absorptance spectra change with the incidence angle like in the case of vacuum (*n* = 1) as the embedding medium. As already shown for the normal incidence configuration, the interparticle coupling diminishes with increasing interparticle gap and at 64 nm, the particles behave like isolated particles for both *n* = 1 or 1.33. In the Kretschmann configuration also, the interparticle coupling becomes negligible in that case, as indicated by a blue-shifted plasmon band relative to the strongly coupled case.

## Conclusions

In this work we have elucidated the plasmonic lattice resonances in Au and Ag nanoparticle arrays in both the direct normal incidence and the Kretschmann (i.e. ATR) configuration, by using numerical models validated by experimental data. For 20 nm nanoparticles, it is shown that the interparticle coupling is almost entirely dominated by near-field coupling with a very weak contribution from radiative coupling. The damping mechanism of the plasmons in the nanoparticles at resonance changes as the interparticle coupling becomes stronger and radiative damping starts dominating. These changes in the plasmon relaxation mechanism have direct consequences for light-driven processes where plasmonic hot-electrons are important, as hot electrons are generated by such non-radiative damping. The per-particle absorption cross-section in a close packed nanoparticle or film is significantly suppressed in comparison to an isolated nanoparticle. This effect is stronger in a dielectric medium in comparison to vacuum, as the plasmonic coupling is stronger in the presence of a dielectric. It is also clear that for close packed films, while the far-field optical properties in normal incidence are independent of polarization due to rotational symmetry, the near-field properties are strongly dependent on the direction of polarization. Although individual Ag nanoparticles have significantly stronger near-field enhancement than Au nanoparticles, when they are closely packed as a 2D array or film, the differences become much smaller. The lattice resonance in plasmonic nanoparticle films in the Kretschmann configuration is excited by the evanescent field with strong absorptance and near-field enhancement. The many features of the far-field optical spectra in the Kretschmann configuration have been resolved by the comparison with the near-field spectra. It was found that under suitable conditions (for example 60° or 75° angle of incidence for Au nanoparticles), close to 100% absorbance with a broad band can be achieved. The results reported herein show the ideal conditions under which the normal and the Kretschmann configuration can be exploited in light-driven applications. In practice, while the normal incidence configuration is ubiquitous, the Kretschmann configuration is used only in dedicated niche applications (e.g. ATR-FTIR). With zero transmission losses, the Kretschmann configuration is particularly advantageous in sensing applications. Use of optical fibres, where light propagates by total internal reflection, can also be interesting in such applications. The fast decaying evanescent field also ensures that the light signal interacts only with the environment close to the sensing platform. The Kretschmann configuration can also be promising for efficient light management in photocatalytic reactors. Similarly, the near-field profiles and the near-field spectra shows that the lattice plasmon resonance results in strong field enhancements also in the Kretschmann configuration. These results are important for surface enhanced spectroscopy in the ATR configuration.

## Supplementary Information

Supplementary Information.

## Data Availability

The datasets used and/or analyzed during the current study are available from the corresponding author on reasonable request.

## References

[1] Kravets VG, Kabashin AV, Barnes WL, Grigorenko AN (2018). Plasmonic surface lattice resonances: A review of properties and applications. Chem. Rev..

[2] Borah R, Verbruggen SW (2019). Coupled plasmon modes in 2D gold nanoparticle clusters and their effect on local temperature control. J. Phys. Chem. C.

[3] Lee M (2015). Aluminum nanoarrays for plasmon-enhanced light harvesting. ACS Nano.

[4] Atwater HA, Polman A (2010). Plasmonics for improved photovoltaic devices. Nat. Mater..

[5] Lim SY (2020). Tailor-engineered plasmonic single-lattices: Harnessing localized surface plasmon resonances for visible-NIR light-enhanced photocatalysis. Catal. Sci. Technol..

[6] Borah R (2021). Selectivity in the ligand functionalization of photocatalytic metal oxide nanoparticles for phase transfer and self-assembly applications. Chem. Eur. J..

[7] Matricardi C (2018). Gold nanoparticle plasmonic superlattices as surface-enhanced Raman spectroscopy substrates. ACS Nano.

[8] Pang JS (2019). Tunable three-dimensional plasmonic arrays for large near-infrared fluorescence enhancement. ACS Appl. Mater. Interfaces.

[9] Zvagelsky R, Chubich D, Pisarenko A, Bedran Z, Zhukova E (2021). Plasmonic metasurfaces as surface-enhanced infrared absorption substrates for optoelectronics: Alq3 thin-film study. J. Phys. Chem. C.

[10] Borah R (2022). Self-assembled ligand-capped plasmonic Au nanoparticle films in the Kretschmann configuration for sensing of volatile organic compounds. ACS Appl. Nano Mater..

[11] Piltan S, Sievenpiper D (2018). Plasmonic nano-arrays for enhanced photoemission and photodetection. J. Opt. Soc. Am. B JOSAB.

[12] Wang L, Hasanzadeh Kafshgari M, Meunier M (2020). Optical properties and applications of plasmonic-metal nanoparticles. Adv. Funct. Mater..

[13] Chekini M (2015). Fluorescence enhancement in large-scale self-assembled gold nanoparticle double arrays. J. Appl. Phys..

[14] Xu Y (2019). Optical refractive index sensors with plasmonic and photonic structures: Promising and inconvenient truth. Adv. Opt. Mater..

[15] Linic S, Christopher P, Ingram DB (2011). Plasmonic-metal nanostructures for efficient conversion of solar to chemical energy. Nat. Mater..

[16] Boles MA, Engel M, Talapin DV (2016). Self-assembly of colloidal nanocrystals: From intricate structures to functional materials. Chem. Rev..

[17] Talapin DV (2009). Quasicrystalline order in self-assembled binary nanoparticle superlattices. Nature.

[18] Benkovičová M (2019). Tailoring the interparticle distance in Langmuir nanoparticle films. Phys. Chem. Chem. Phys..

[19] Oliveira JP (2019). Quantification of inter-particle spacing caused by thiol self-assembled monolayers using transmission electron microscopy. Plasmonics.

[20] Tao A, Sinsermsuksakul P, Yang P (2007). Tunable plasmonic lattices of silver nanocrystals. Nat. Nanotechnol..

[21] Grzelczak M, Vermant J, Furst EM, Liz-Marzán LM (2010). Directed self-assembly of nanoparticles. ACS Nano.

[22] Bansmann J (2007). Controlling the interparticle spacing of Au–Salt loaded micelles and Au nanoparticles on flat surfaces. Langmuir.

[23] Mejía-Salazar JR, Oliveira ON (2018). Plasmonic biosensing. Chem. Rev..

[24] Bastús NG, Merkoçi F, Piella J, Puntes V (2014). Synthesis of highly monodisperse citrate-stabilized silver nanoparticles of up to 200 nm: Kinetic control and catalytic properties. Chem. Mater..

[25] Lau CY (2011). Enhanced ordering in gold nanoparticles self-assembly through excess free ligands. Langmuir.

[26] Huang S, Minami K, Sakaue H, Shingubara S, Takahagi T (2004). Effects of the surface pressure on the formation of Langmuir–Blodgett monolayer of nanoparticles. Langmuir.

[27] Ross MB, Ku JC, Blaber MG, Mirkin CA, Schatz GC (2015). Defect tolerance and the effect of structural inhomogeneity in plasmonic DNA–nanoparticle superlattices. PNAS.

[28] Sani E, Dell’Oro A (2014). Optical constants of ethylene glycol over an extremely wide spectral range. Opt. Mater..

[29] Johnson PB, Christy RW (1972). Optical constants of the noble metals. Phys. Rev. B.

[30] Palik ED (1998). Handbook of Optical Constants of Solids.

[31] Borah R, Verbruggen S (2022). Effect of size distribution, skewness and roughness on the optical properties of colloidal plasmonic nanoparticles. Colloids Surf. A.

[32] Kreibig U, Vollmer M (1995). Optical Properties of Metal Clusters.

[33] Barrow SJ, Wei X, Baldauf JS, Funston AM, Mulvaney P (2012). The surface plasmon modes of self-assembled gold nanocrystals. Nat. Commun..

[34] Brown AM, Sundararaman R, Narang P, Goddard WA, Atwater HA (2016). Nonradiative plasmon decay and hot carrier dynamics: Effects of phonons, surfaces, and geometry. ACS Nano.

[35] Valenti M (2017). Hot carrier generation and extraction of plasmonic alloy nanoparticles. ACS Photonics.

[36] Borah R, Verbruggen SW (2020). Silver-gold bimetallic alloy versus core-shell nanoparticles: Implications for plasmonic enhancement and photothermal applications. J. Phys. Chem. C.

[37] Prodan E, Lee A, Nordlander P (2002). The effect of a dielectric core and embedding medium on the polarizability of metallic nanoshells. Chem. Phys. Lett..

[38] Zhou X (2011). Effects of dielectric core and embedding medium on plasmonic coupling of gold nanoshell arrays. Solid State Commun..

[39] Zakomirnyi VI (2019). Collective lattice resonances in arrays of dielectric nanoparticles: A matter of size. Opt. Lett. OL.

[40] Khurgin J, Tsai W-Y, Tsai DP, Sun G (2017). Landau damping and limit to field confinement and enhancement in plasmonic dimers. ACS Photonics.

[41] Huang Y (2016). Hybridized plasmon modes and near-field enhancement of metallic nanoparticle-dimer on a mirror. Sci. Rep..

[42] Zong C (2019). Plasmon-enhanced stimulated Raman scattering microscopy with single-molecule detection sensitivity. Nat. Commun..

[43] Rahmani M (2013). Plasmonic nanoclusters with rotational symmetry: Polarization-invariant far-field response vs changing near-field distribution. ACS Nano.

[44] Pluchery O, Vayron R, Van K-M (2011). Laboratory experiments for exploring the surface plasmon resonance. Eur. J. Phys..

[45] Mosleh M, Ranjbaran M, Hamidi SM (2021). Trace of evanescent wave polarization by atomic vapor spectroscopy. Sci. Rep..

[46] Martin-Fernandez ML, Tynan CJ, Webb SED (2013). A ‘pocket guide’ to total internal reflection fluorescence. J. Microsc..

[47] Ras RHA, Schoonheydt RA, Johnston CT (2007). Relation between s-polarized and p-polarized internal reflection spectra: Application for the spectral resolution of perpendicular vibrational modes. J. Phys. Chem. A.

[48] Bruno OP, Fernandez-Lado AG (2017). Rapidly convergent quasi-periodic Green functions for scattering by arrays of cylinders—Including Wood anomalies. Proc. R. Soc. A Math. Phys. Eng. Sci..

[49] Fernandez-Lado AG (2020). Wave-Scattering by Periodic Media.

[50] Darweesh AA, Bauman SJ, Debu DT, Herzog JB (2018). The role of Rayleigh-wood anomalies and surface plasmons in optical enhancement for nano-gratings. Nanomaterials.

[51] Manera MG, Colombelli A, Taurino A, Martin AG, Rella R (2018). Magneto-Optical properties of noble-metal nanostructures: Functional nanomaterials for bio sensing. Sci. Rep..

[52] Kravets VG, Schedin F, Grigorenko AN (2008). Extremely narrow plasmon resonances based on diffraction coupling of localized plasmons in arrays of metallic nanoparticles. Phys. Rev. Lett..

