# Supplementary material for: Plasmon resonance of gold and silver nanoparticle arrays in the Kretschmann (attenuated total reflectance) vs. direct incidence configuration
Source: Sci Rep. 2022 Sep 21;12:15738. doi: 10.1038/s41598-022-20117-7 (PMC9492765; doi:10.1038/s41598-022-20117-7)
Supplement: Supplementary file 1 — Supplementary Information. [file 41598_2022_20117_MOESM1_ESM.docx]

**Plasmon Resonance of Gold and Silver Nanoparticle Arrays in the Kretschmann (Attenuated Total Reflectance) *vs*. Direct Incidence Configuration**

Rituraj Borah^1,3^, Rajeshreddy Ninakanti^1,2,3^, Sara Bals^2,3^, Sammy W. Verbruggen^1,3✉^

^1^Sustainable Energy, Air & Water Technology (DuEL), Department of Bioscience Engineering, University of Antwerp, Groenenborgerlaan 171, 2020 Antwerp (Belgium)

^2^Electron Microscopy for Material Science (EMAT) Department of Physics, University of Antwerp, Groenenborgerlaan 171, 2020 Antwerp (Belgium)

^3^NANOlab Center of Excellence, University of Antwerp, Groenenborgerlaan 171, 2020 Antwerp (Belgium)

^✉^email: [sammy.verbruggen@uantwerpen.be](mailto:sammy.verbruggen@uantwerpen.be)

**Computational electromagnetic modeling**

For the electromagnetic modeling, the Maxwell’s equation derived for the frequency domain as given below was solved numerically by an FEM solver COMSOL Multiphysics:

$\nabla\times({\mu_{r}}^{-1}\nabla\times\boldsymbol{E})-{k_{o}}^{2}(\varepsilon_{r}^{'}-j\frac{\sigma_{e}}{\omega\varepsilon_{o}})\boldsymbol{E}=0$ (S.1)

In equation (S.1), *μ_r_*, *ε_o_* and *σ_e_* are material properties namely relative permeability, permittivity of free space and electrical conductivity respectively, and *k_o_* denotes the wavenumber. For both Au and Ag, the relative permeability is assumed to be 1. Now, $\varepsilon_{r}^{'}$ is the real part of the complex relative permittivity (or dielectric constant) and importantly, *σ_e_* is directly connected to the imaginary part of the dielectric constant as *σ_e_* = $\varepsilon_{r}^{''}$ *ω,* where $\varepsilon_{r}^{''}$ is the imaginary part of the dielectric constant. Thus, in COMSOL, the complex dielectric constants completely defines the material optical properties. The conductivity term *σ_e_* in equation (S.1) does not need to be specified. In literature, optical properties are reported as both dielectric constants and refractive index, which are related by well-known mathematical expressions. For the numerical solution of Equation (S.1) for an infinite nanoparticle array, an unit cell was constructed as the computational domain in Figure 1 (d) (main text) and Figure S1, S2. To test the accuracy of the periodic boundary conditions two different unit cell configurations were compared. As shown in Figure S1 and S2, Floquet periodic boundary conditions were imposed on the side walls. Both the rectangular and hexagonal unit cells can be used for the computations as the spectral intensities are the same for both the geometries as shown in Figure 1 (e). Importantly, adaptive meshing is required for periodic boundary condition to work accurately. Thus, an exact meshing scheme on the opposite side walls was imposed. PML (perfectly matched layer) domains were constructed at the top and the bottom so that these walls are perfectly absorbing and do not reflect any incident wave. In the total field mode in COMSOL, an incident field is excited at the top with an exit port at the bottom. After adaptive meshing of the walls, the rest of the computational domains was discretized by tetrahedral elements with refinement in the narrow/corner regions. After the solution for the electric field, the magnetic field can be directly obtained from the electric field by the relationship:

$\boldsymbol{H}= \frac{1}{Z}k\times\boldsymbol{E} =\sqrt{\frac{\varepsilon}{\mu}}k\times\boldsymbol{E}$ (S.2)

or,

$\boldsymbol{H}=\frac{1}{Z_{o}}\sqrt{\frac{\varepsilon_{r}}{\mu_{r}}}k\times\boldsymbol{E}$ (S.3)

Where, *Z* is the complex impedance of the medium/material. In equation S.2, *ε* and *μ* are the permittivity of the medium. For the consistency with equation (S.1), the impedance is expressed in terms of relative permittivity (*ε_r_*), relative permeability (*μ_r_*) and free space impedance (*Z_o_*).

From the numerical solution of the electric field and magnetic field, the optical intensities are obtained by mathematical post-processing. The energy absorbed per unit time, $W_{abs}$, by the nanoparticles can be calculated by both the following equations:

$W_{abs}=\frac{1}{2}\iiint_{V} Re [(\sigma\boldsymbol{E}+j\omega\boldsymbol{D}).\boldsymbol{E}^{*}+j\omega\boldsymbol{B}.\boldsymbol{H}^{*}]dV$ (S.4)

$W_{abs}=\frac{1}{2}∯_{s} Re [\boldsymbol{E}\times\boldsymbol{H}^{*}].ndS$ (S.5)

The volume integration and the surface integration in the above equations are applied over the volume and outer surface of the nanoparticle respectively. The superscript * and ***D*** stand for complex conjugate and displacement currents respectively. Like the integration of the Poynting vector over nanoparticle surface performed in equation (S.5), the transmittance or reflectance can also be obtained by the integration of the Poynting vector at the inlet/exit port. Similarly, COMSOL facilitates the direct computation of the complex reflectance (or reflectivity) from the s11 parameter.

**Supplementary figures**


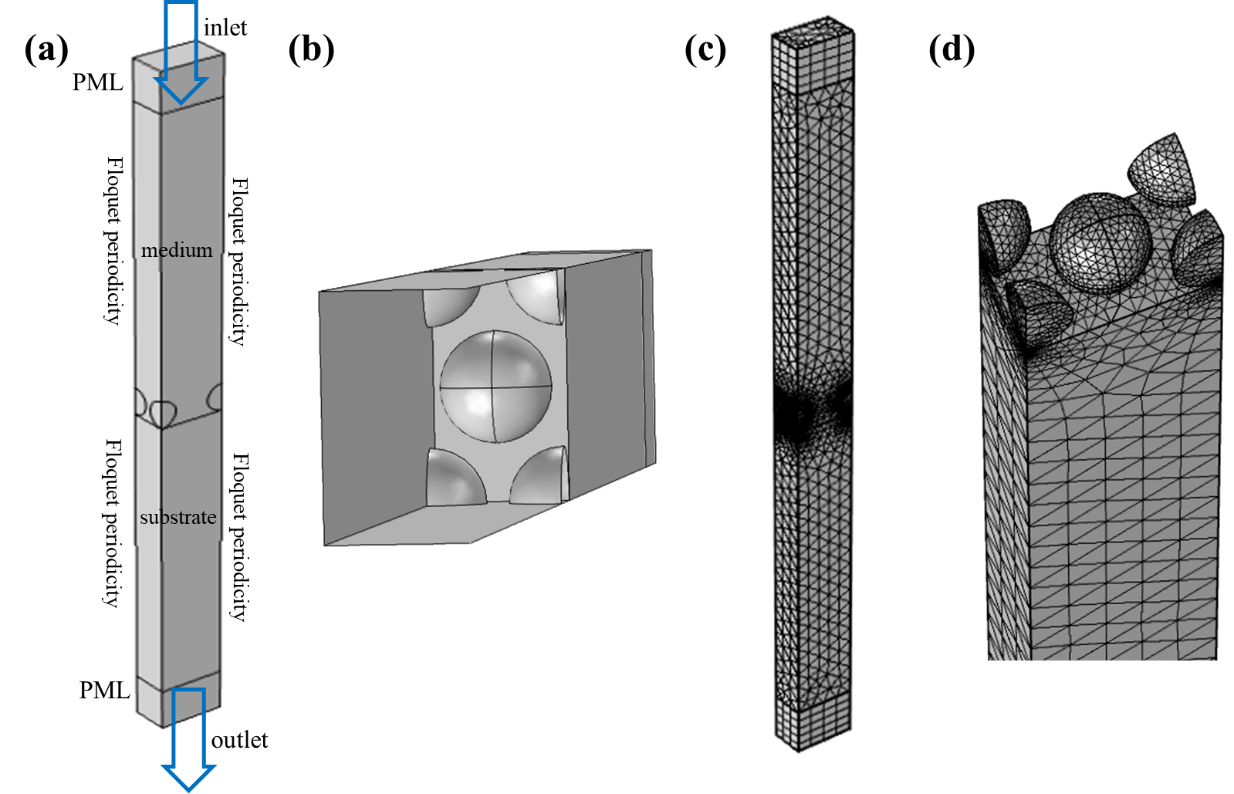


**Figure S1**. Size distribution of Au nanoparticles (average diameter: 9.56 nm from 45 data points, average interparticle gap: 2 nm from 18 data points).


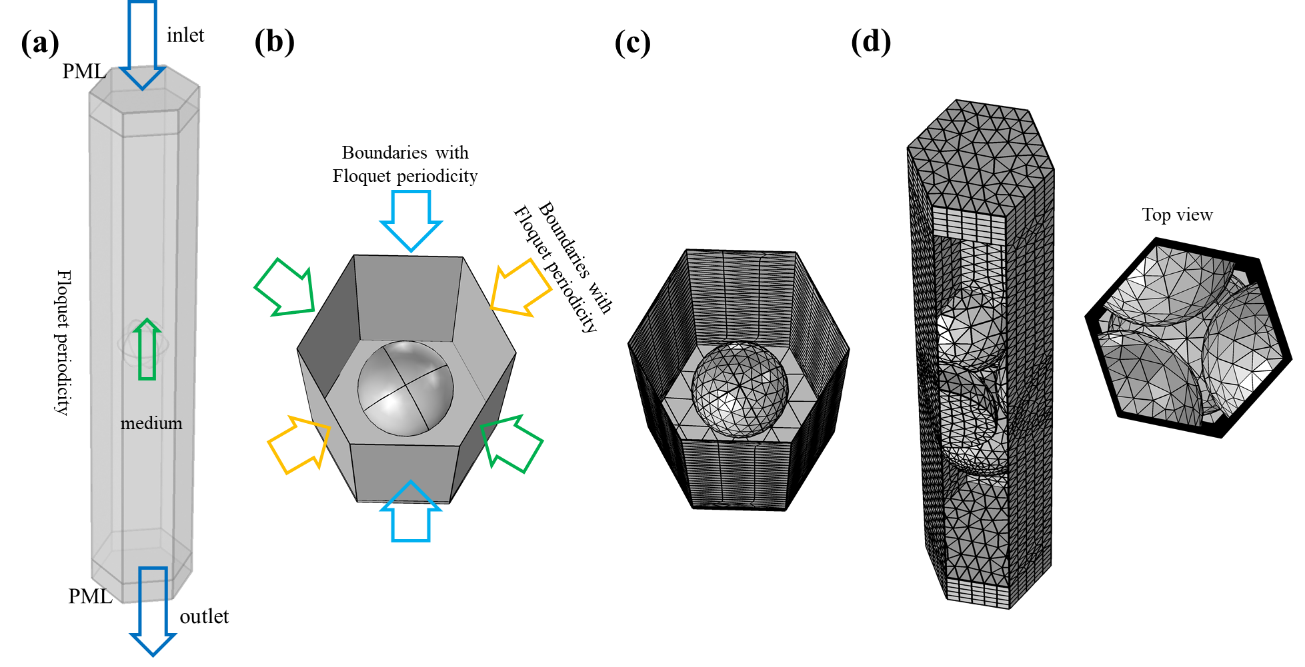


**Figure S2**. Size distribution of Au nanoparticles (average diameter: 9.56 nm from 45 data points, average interparticle gap: 2 nm from 18 data points).


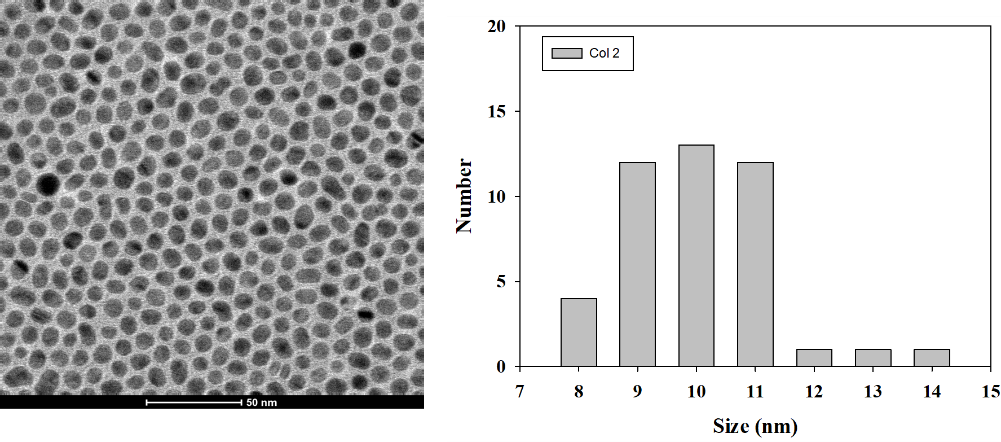


**Figure S3**. Size distribution of Au nanoparticles (average diameter: 9.56 nm from 45 data points, average interparticle gap: 2.1 nm from 18 data points).


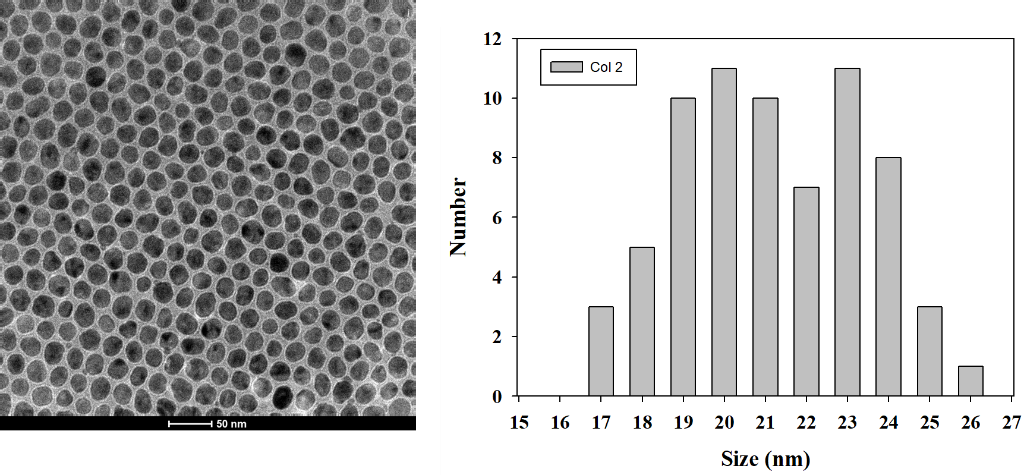


**Figure S4**. Size distribution of Ag nanoparticles (average diameter: 20.76 nm from 70 data points, average interparticle gap: 2.98 nm from 20 data points).


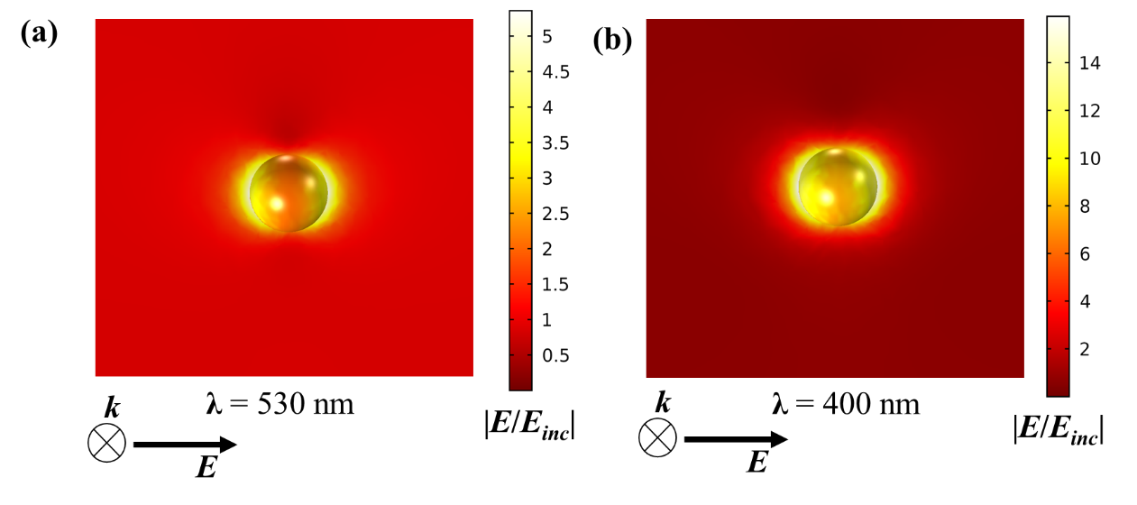


**Figure S5**. Near-field enhancement of 20 nm large Au and Ag nanoparticles in dielectric medium (*n* = 1.33) when isolated.

**
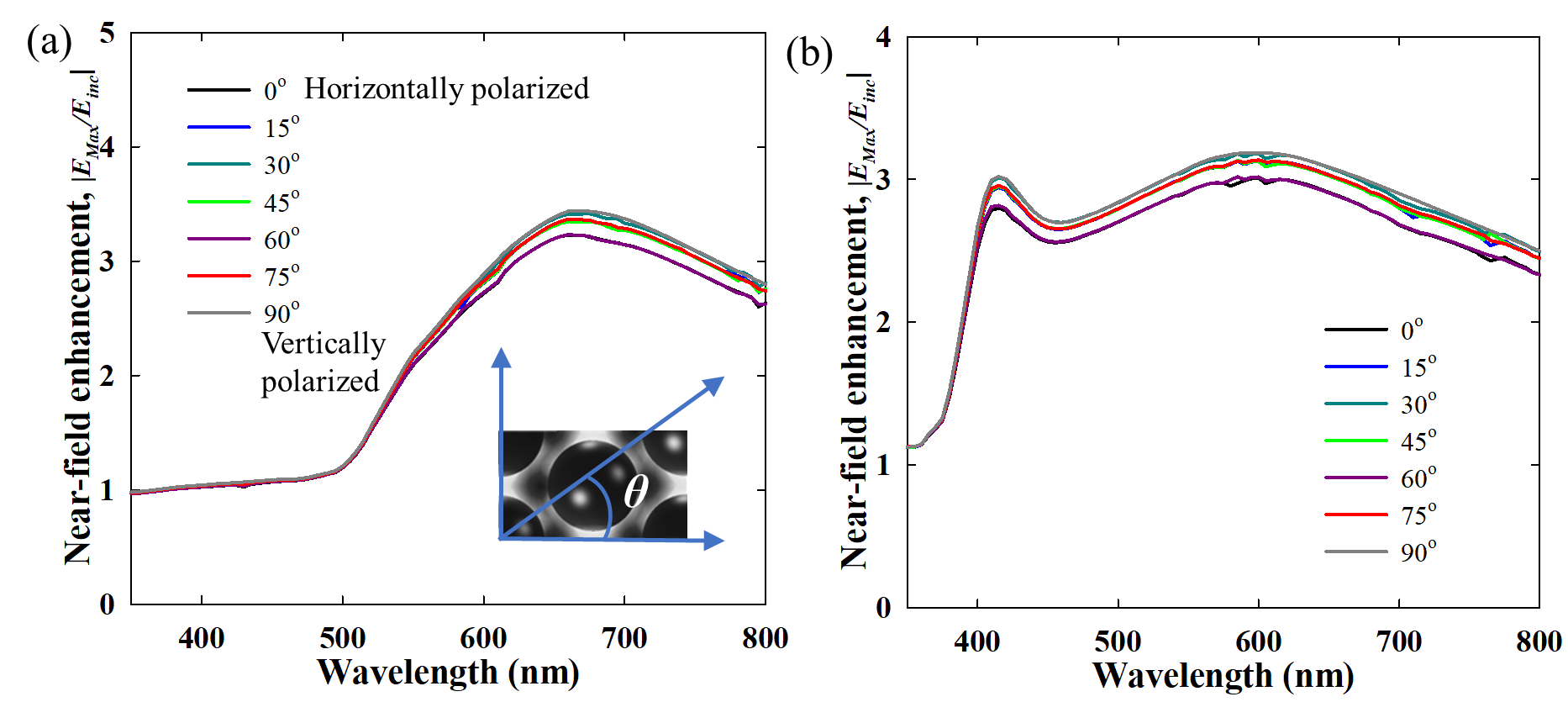
**

**Figure S6**. The average near-enhancement spectra of Au (a) and Ag (b) nanoparticle assemblies (shown only for interparticle gap of 1 nm) at different polarizations indicated by angle, *φ* and normal direct incidence. Horizontal polarization is *φ* = 0^o^ and vertical polarization is *φ* = 90^o^. (embedding medium, *n* = 1.33)


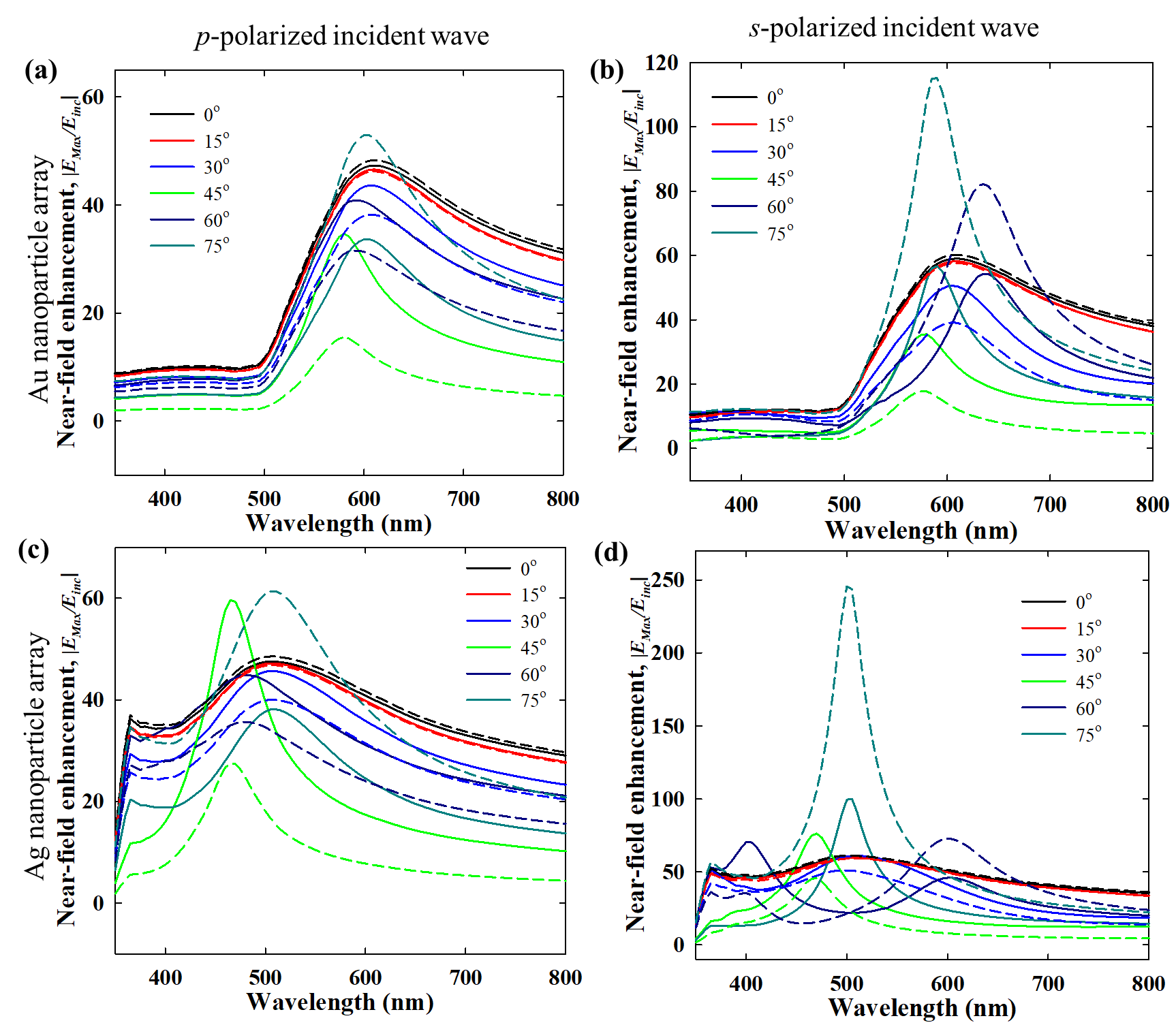


**Figure S7.** Near-field maximum spectra of close packed 20 nm Au (a, b) and Ag (c, d) nanoparticle films in the Kretschmann *i.e*. ATR configuration for *p*- and *s*-polarized (or TM and TE polarized) incident wave, and varying incident angle (*n* = 1.5 for the denser medium, *n* = 1 for the lighter medium and interparticle gap: 1 nm). Legend: for each color from black to cyan, the solid lines represent near-field enhancement *w.r.t*. the incident field (without the Kretschmann configuration) and the dotted lines represent near-field enhancement *w.r.t* the background evanescent field in the absence of the nanoparticles.


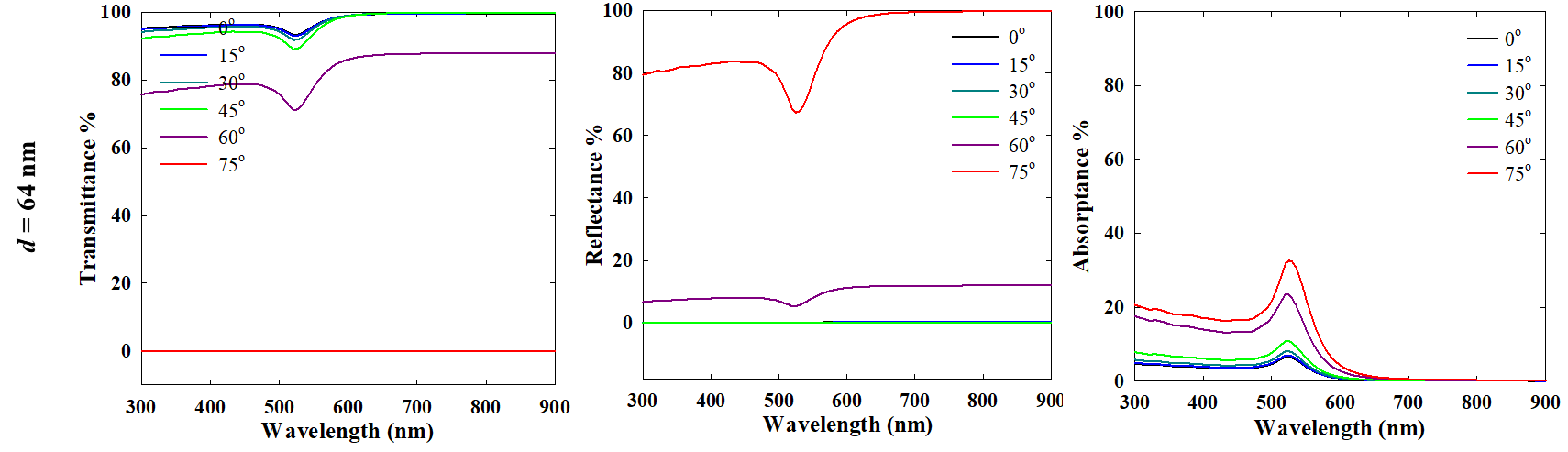


**Figure S8.** Transmittance, reflectance and absorptance spectra of 20 nm Au nanoparticle array with 64 nm interparticle distance in the Kretschmann *i.e*. ATR configuration for *p*-polarized incident wave, and varying incident angle (*n* = 1.5 for the denser medium, *n* = 1.33 for the lighter medium).
